# Impacts of heatwaves and cold spells on glaucoma in rural China: a national cross-sectional study

**DOI:** 10.1007/s11356-023-25591-8

**Published:** 2023-02-03

**Authors:** Ai Zhang, Qihua Wang, Xueli Yang, Yuanyuan Liu, Jiayu He, Anqi Shan, Naixiu Sun, Qianfeng Liu, Baoqun Yao, Fengchao Liang, Ze Yang, Xiaochang Yan, Shaoye Bo, Yang Liu, Hongjun Mao, Xi Chen, Nai-jun Tang, Hua Yan

**Affiliations:** 1 Department of Occupational and Environmental Health, School of Public Health, Tianjin Medical University, Tianjin, China; 2 Tianjin Key Laboratory of Environment, Nutrition, and Public Health, Tianjin Medical University, Tianjin, China; 3 Department of Ophthalmology, Tianjin Medical University General Hospital, Tianjin Key Laboratory of Ocular Trauma, Laboratory of Molecular Ophthalmology, Tianjin Medical University, Tianjin, China; 4 Beijing Tsinghua Changgung Hospital, School of Clinical Medicine, Tsinghua University, Beijing, China; 5 Tianjin Key Laboratory of Urban Transport Emission Research, College of Environmental Science and Engineering, Nankai University, Tianjin, China; 6 School of Public Health and Emergency Management, Southern University of Science and Technology, Shenzhen, China; 7 National School of Development, Peking University, Beijing, China; 8 China Foundation for Disabled Persons, Dongcheng District, Beijing, China; 9 Gangarosa Department of Environmental Health, Rollins School of Public Health, Emory University, Atlanta, GA USA; 10 School of Medicine, Nankai University, Tianjin, China

**Keywords:** Heatwaves, Cold spells, Glaucoma, PM_2.5_, PACG, Cross-sectional study

## Abstract

China faces increasing health risks from climate change. The structure and function of the eye and vision were affected by extreme heat and cold. The study aimed to evaluate the impacts of heatwaves and cold spells on glaucoma. A national cross-sectional study of the Rural Epidemiology for Glaucoma (REG-China) was conducted in ten provinces of China, and 36,081 adults aged 40 years or more were included. Glaucoma signs were assessed via a standard examination. A total of 15 heatwave definitions, based on intensity (95th to 99th percentiles of temperature distribution) and duration (≥2 days, 3 days, and 4 days), were used to quantify heatwave effects, and 6 cold spell definitions were defined based on threshold temperature percentile (5th and 10th) and duration (3 days, 5 days, and 9 days). Multivariable-adjusted logistic regression models paired with interaction analysis were performed to investigate the impacts of heatwaves and cold spells on glaucoma, and the dose-response relationships were assessed using a restricted cubic spline (RCS) model. Subgroup analysis was conducted stratified by gender, age, smoking status, occupation, and family history of glaucoma. The overall prevalence of glaucoma was 2.1% (95% CI 1.94–2.25%). Higher heatwaves were significantly correlated with higher OR of glaucoma, with the OR (95% CI) ranging from 1.014 (1.009, 1.018) to 1.090 (1.065, 1.115) by different definitions. Glaucoma was affected by heatwaves more strongly than by cold spells. The effects of both heatwaves and cold spells were higher in males than females and in smokers than nonsmokers. These results of the present study evoked the attention of prospective research to elucidate the relationship between extreme temperatures and eye diseases.

**Supplementary Information:**

The online version contains supplementary material available at 10.1007/s11356-023-25591-8.

## Introduction

China faces growing health risks from climate change, but also the opportunity to address them and protect the health of populations. If climate change is not responded quickly and adequately, it will impact lives and livelihoods at a rapid pace (Cai et al. 2021). Extreme weather events (EWEs) (e.g., heatwaves and cold spells) were associated with high morbidity through a range of physiological mechanisms (Kephart et al. 2022). Extreme heat and cold temperatures also affect the structure and function of the eye and vision (Jaki Mekjavic et al. 2021). It is possible that extreme temperatures and continued exposure to air pollution place additional burdens on eye health.

Glaucoma is a heterogeneous group of diseases characterized by cupping of the optic nerve head and visual-field damage. It is the most frequent cause of irreversible blindness worldwide (Jonas et al. 2017). Intraocular pressure (IOP) is the only modifiable risk factor known to exist. Various internal and external factors, including systemic blood pressure (Klein et al. 2005, Langman et al. 2005) and ambient temperature (Liao et al. 2022, Morettin et al. 2021, Terauchi et al. 2021), have an impact on IOP. Older age, non-white race, and family history of glaucoma are other important risk factors (Stein et al. 2021). Glaucoma Rural Epidemiology in China (REG-China) was performed in our previous study, which was the first nationwide epidemiological study conducted in rural areas. In the REG-China study, estimating the prevalence and risk factors for glaucoma at the national level was our main concern, along with assessing disease burden and informing different strategies to enhance public health programs for eye disease prevention and treatment (Liu et al. 2022). We expected to evaluate risk factors furtherly from ambient environment for glaucoma. In our previous study, we found that long-term exposure to air pollution was associated with glaucoma and diabetic retinopathy based on the REG-China study (Shan et al. 2021, Yang et al. 2021).

The impacts of heatwaves and cold spells on ophthalmology diseases were relatively limited. Some researchers believed that higher ambient temperature is a risk factor for the development of glaucoma (Shan et al. 2021). Some studies have shown that IOP tends to be higher on cold days than on hot days. IOP was negatively correlated with ambient temperature and sunshine time (Liao et al. 2022). It is recommended that people with narrow anterior chamber angles or a family history of acute primary angle closure remain indoors during the hot summer (Zhong et al. 2021). Extreme climatic events, such as heatwaves and cold spells, can pose ongoing health risks.

To understand the impact of heatwaves and cold spells on glaucoma, given the limited research in this area and the biological plausibility of an association, in the current national study, we aimed to elucidate the impacts of heatwaves and cold spells on glaucoma in the rural population of China. Our findings could provide additional insights relevant to glaucoma, which would help improve prevention and control strategies.

## Material and methods

### Study population

The study population was from REG-China. REG-China examined the rural population in 10 provinces, autonomous areas, and municipalities across the country. The nationally representative population was sampled and registered with the use of a multistage stratified cluster sampling technique. The specifics of the study’s design were covered in other places as well as the Supplemental Methods (Liu et al. 2022, Shan et al. 2021, Yang et al. 2021).

**Fig. 1:**
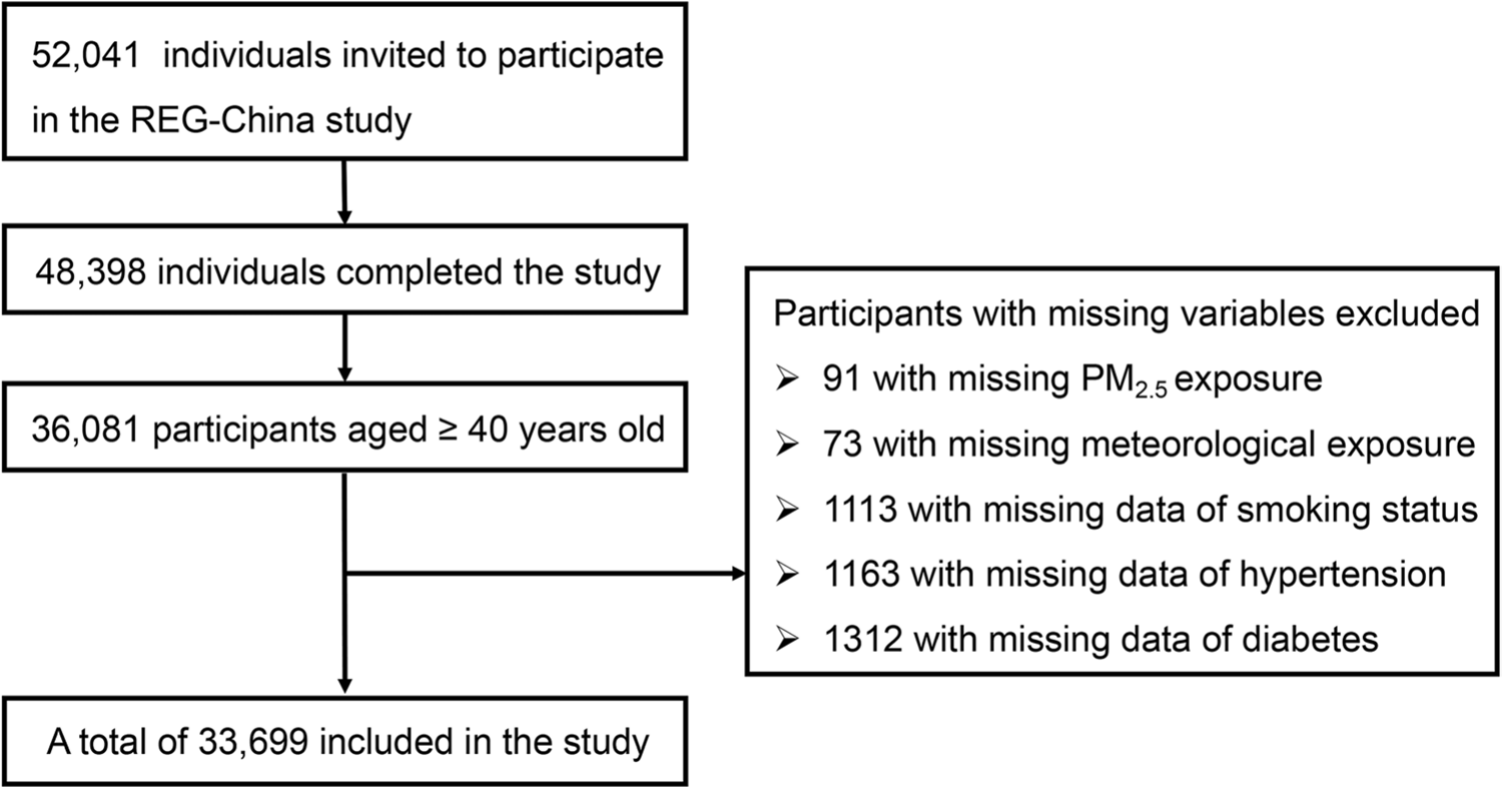
The flowchart for inclusion and exclusion of study participants. The study population was based on the Rural Epidemiology for Glaucoma in China (REG-China). This study is a cross-sectional analysis of a nationally representative sample of people from ten rural Chinese provinces, autonomous regions, and municipalities

From June 2017 to October 2018, 52,041 people were recruited for the REG-China research. In the present analysis, 36,081 participants aged ≥40 years old were selected from the REG-China study. Among the 36,081 participants, 33,699 participants with health data were included in the further analysis (Fig. 1). The research project was approved by the Tianjin Medical University Research Ethics Committee, and we conducted all survey methods in accordance with the Helsinki Declaration Principles. Written informed consent of each subject was obtained prior to the study.

### Questionnaires and eye examination

Using thorough interviewer-administered questions, the demographic information, history of ocular illnesses, and family history of glaucoma were gathered. Each participant underwent a standard ophthalmologic examination, which included measuring IOP with noncontact tonometry and performing distance visual acuity testing, best-corrected visual acuity testing, direct ophthalmoscopy, slit-lamp microscopy, and slit-lamp biomicroscopy with a 90D convex lens without pupil dilation (Foster et al. 2002, Foster et al. 2000). The details of eye examination and quality control were described elsewhere and also in the Supplemental Methods (Liu et al. 2022).

### Case definition

Glaucoma was diagnosed by ophthalmologists based on three levels of evidence claimed by the International Society of Geographical and Epidemiological Ophthalmology (ISGEO), and based on ISGEO criteria, glaucoma was categorized into three categories (detailed in the Supplementary Methods). Primary open-angle glaucoma (POAG) refers to an eye with glaucoma symptoms but no angle closure on gonioscopy and no discernible secondary etiology. According to the International Glaucoma Association, primary angle-closure glaucoma (PACG) was identified in conjunction with acute start of IOP, persistent acute angle-closure or chronic IOP elevation indications, or ocular signs after filtration surgery, removal of the peripheral iris, or laser removal of the peripheral iris. Childhood glaucoma in its primary and tertiary forms was referred to as congenital glaucoma (CG) (Liang et al. 2011, Liang et al. 2009, Papadopoulos et al. 2007, Yeung &Walton 2010).

### Environment exposure assessment

#### Air pollution

PM_2.5_ concentrations were estimated at 1 km spatial resolution using an established satellite-based spatiotemporal model, and procedures in detail have been provided elsewhere (Shan et al. 2021, Yang et al. 2021). As shown in Supplemental Methods, each participant’s residential addresses were geocoded to their respective longitudes and latitudes. PM_2.5_ exposure levels were matched to participants’ home addresses by latitude and longitude. Average exposure levels of PM_2.5_ from 2007 to 2016 were calculated as the long-term exposure level used in the study.

In a nutshell, the Moderate Resolution Imaging Spectrometer (MODIS) satellite operated by the US National Aeronautics and Space Administration (NASA) was the source of the aerosol optical depth (AOD) product obtained using the Multi-Angle Implementation of Atmospheric Correction (MAIAC) method, which uses a machine learning algorithm to estimate PM_2.5_ concentrations by linking AOD indicators to other indicators such as meteorology, road networks, land cover indices, and air pollution emissions (Liang et al. 2020, Randles et al. 2017, Xiao et al. 2018).

#### Temperature

The temperature during the study period was calculated by ERA5 (European Centre for Medium-Range Weather Forecasts reanalysis v5). ERA5 provides hourly data at 0.25° resolution (approximately 28 km) on regular latitude-longitude grids, currently during the satellite period (1979 to present) (Hersbach et al. 2020, Hoffmann & Spang 2022). We retrieved ERA5 single-level hourly data for 2 m (meter) air temperature for the period 2007–2016 from the Copernicus Climate Data Store utilizing a Python script suggested by the European Centre for Medium-Range Weather Forecasts. “2 m air temperature” refers to the air temperature at 2 m height. After decoding (using the Climate Data Operators tool) and average processing, the daily mean temperature of grid points was obtained. In addition, to match the ERA5 grid points to participants’ home addresses, grid points were interpolated to the study points according to the latitude and longitude of home addresses using a linear interpolation method.

#### Heatwaves

Studies have found that the relative risk of mortality started to increase around the 95th percentile of temperature, increased sharply at the 97th percentile, and rose alarmingly at the 99th percentile. They also proved that the model using heatwaves with a duration of ≥2 days produced a better model fit than those using a duration of ≥3 days or ≥4 days. Therefore, as shown in Table 1, we used intensity (95th, 96th, 97th, 98th, or 99th percentile of mean temperature distribution from January 1, 2007, to December 31, 2016) and duration (2 days, 3 days, and 4 days) to determine heatwaves. The mean temperature was calculated by ERA5, and average temperatures are used because they are better than maximum or minimum temperatures when evaluating the health effects of heatwaves (Tong et al. 2015, Tong et al. 2014, Xu et al. 2018).

**Table 1:** Heatwave definitions

Heatwaves	Abbreviations	Specific definition	Threshold temperature (°C)	Average number of times per person (times)	Average number of days per person (days)
Heatwave 01	HW01	95^th^ & ≥2 days	28.2	28.6	130.9
Heatwave 02	HW02	95^th^ & ≥3 days	28.2	17.9	109.7
Heatwave 03	HW03	95^th^ & ≥4 days	28.2	12.4	93.5
Heatwave 04	HW04	96^th^ & ≥2 days	29.3	23.2	102.5
Heatwave 05	HW05	96^th^ & ≥3 days	29.3	15.1	86.4
Heatwave 06	HW06	96^th^ & ≥4 days	29.3	10.1	71.8
Heatwave 07	HW07	97^th^ & ≥2 days	30.0	17.4	73.0
Heatwave 08	HW08	97^th^ & ≥3 days	30.0	11.3	60.7
Heatwave 09	HW09	97^th^ & ≥4 days	30.0	7.5	50.0
Heatwave 10	HW10	98^th^ & ≥2 days	31.1	12.2	46.1
Heatwave 11	HW11	98^th^ & ≥3 days	31.1	7.0	35.8
Heatwave 12	HW12	98^th^ & ≥4 days	31.1	4.8	29.1
Heatwave 13	HW13	99^th^ & ≥2 days	31.9	5.9	20.8
Heatwave 14	HW14	99^th^ & ≥3 days	31.9	2.8	14.6
Heatwave 15	HW15	99^th^ & ≥4 days	31.9	1.8	11.4

#### Cold spells

The temperature used to define the cold spells was calculated by ERA5. In this study, cold spells were defined through the following three steps (Jing-Bei & Cholaw 2011, Ma et al. 2013, Wang et al. 2016). First, select 1 October to 30 April (from 2007 to 2016) as the research period of the cold spell. For each participant, the average 2 m temperature of 3 days, 5 days, and 9 days in the moving window centered on the study day was calculated respectively (3 days–2 m temperature, 5 days–2 m temperature, and 9 days–2 m temperature). Second, the daily threshold indices for the fifth and tenth percentiles (5th, 10th) for each participant in the cold spells study period were calculated, respectively. Third, a participant was considered to have had a “cold spell” day if the average temperature was lower than the threshold. These conditions consider both a temperature test and a duration test. According to the above concepts, six different definitions of “cold spells” are obtained, as displayed in Table 2.

**Table 2:** Cold spell definitions

Cold spells	Abbreviations	Threshold percentile	Windows of moving average (days)	Average number of days per person (days)
Cold spell 01	CS01	10	9	199.7
Cold spell 02	CS02	10	5	187.6
Cold spell 03	CS03	10	3	170.2
Cold spell 04	CS04	5	9	75.8
Cold spell 05	CS05	5	5	64.9
Cold spell 06	CS06	5	3	46.9

### Statistical analysis

Demographic characteristics were aggregated using the median and quartile range for continuous variables and frequency and percentage for categorical variables. The Mann-Whitney-Wilcoxon test and Fisher’s exact test were used to make statistical comparisons of the demographic characteristics of the various groups. Multivariable-adjusted logistic regression models were then used to evaluate the potential association between heatwaves, cold spells, and glaucoma. All analyses were adjusted for age (continuous variable), gender (male vs. female), education levels (primary or less vs. middle or high school vs. college or more), occupation (nonfarmer vs. farmer), personal annual income (<30,000 vs. 30,000–80,000 vs. >80,000), regions (east vs. central vs. west), smoking status (never vs. current), hypertension (yes vs. no), diabetes (yes vs. no), IOP (continuous variable), and PM_2.5_ (continuous variable).

We assessed the dose-response relationships between heatwaves, cold spells, and temperature with the risk of IOP, central anterior chamber depth (CACD), and glaucoma using restricted cubic spline (RCS) models (Durrleman & Simon 1989), adjusting for gender, age, regions, education levels, occupation, personal annual income, smoking status, hypertension history, diabetes history, and PM_2.5_ levels in the model. Plotted smooth curves with three knots at the 5th, 50th, and 95th percentiles of heatwave 01 (HW01) (temperature ≥95th and duration ≥ 2 days), cold spell 01 (CS01) (10th and 9 days–2 m temperature), and daily mean temperature.

Subgroup analysis was also conducted stratified by gender (male vs. female), age (40–65/>65), smoking status (never vs. current), occupation (nonfarmer vs. farmer), and family history of glaucoma (yes vs. no). To ascertain if variations in effect estimates in the subgroup analysis were statistically significant, Eq. (1) was employed. Moreover, the statistical interactions of heatwaves, cold spells, and PM_2.5_ were detected by introducing multiplicative terms.

1 $$Z=\ln (Q1)-\ln (Q2)/\sqrt{\textrm{SE}{1}^2+\textrm{SE}{2}^2},$$

where *Q*1 and *Q*2 are the estimated OR values in each subgroup and SE1 and SE2 are the relative standard errors.

We performed several sensitivity studies to evaluate the robustness of our primary conclusions. First, we exclude individuals with cataract surgery (Sun et al. 2017). Second, we excluded participants with chronic diseases (hypertension and diabetes). Third, other meteorological factors could affect the effects of heatwaves and cold spells, so we added relative humidity and air pressure to the model.

R software (version 4.2.1, R Foundation for Statistical Computing, Vienna, Austria) and IBM SPSS Statistics for Windows (version 24.0, IBM Corp., USA) were used for all statistical analyses (version 4.2.1, R Foundation for Statistical Computing, Vienna, Austria). Statistical significance was defined as two-sided *P* < 0.05.

## Results

### General characteristics of population and the prevalence of glaucoma

Table 3 shows the baseline characteristics of the study population. The average age of all participants was 62.27 years, with 39.3% of them being male. Approximately 63.8% of participants had a lower education level. 90.2% of participants were married. 69.7% of participants were farmers, and 89.2% had a personal annual income of less than 30,000. 24.7% of all participants smoked.

**Table 3:** Baseline characteristics of the participants

	Glaucoma	PACG	POAG	Nonglaucoma	Total
Participants (%)	707 (2.10%)	326 (0.97%)	244 (0.72%)	32,992 (97.90%)	33,699
Sex (%)					
Male	283 (40.0%)	118 (36.2%)	115 (47.1%)	12,968 (39.3%)	13,251 (39.3%)
Female	424 (60.0%)	208 (63.8%)	129 (52.9%)	20,022 (60.7%)	20,466 (60.7%)
Age (year)^*a*^	66.9 (10.48)	68.54 (9.78)	65.18 (11.6)	62.17 (11.3)	62.27 (11.28)
Education level (%)					
Primary school or less	482 (70.1%)	226 (70.8%)	159 (67.4%)	20,260 (63.7%)	20,742 (63.8%)
Middle or high school	166 (24.1%)	66 (20.7%)	68 (28.8%)	10,284 (32.3%)	10,450 (32.1%)
College or more	40 (5.8%)	27 (8.5%)	9 (3.8%)	1281 (4.0%)	1321 (4.1%)
Marital status (%)					
Never married	24 (3.4%)	8 (2.5%)	15 (6.2%)	714 (2.2%)	738 (2.2%)
Married/common	605 (86.2%)	279 (86.4%)	201 (83.1%)	29,583 (90.3%)	30,188 (90.2%)
Divorced/widowed	73 (10.4%)	36 (11.1%)	26 (10.7%)	2459 (7.5%)	2532 (7.6%)
Occupation (%)					
Farmer	485 (69.0%)	228 (70.6%)	161 (66.3%)	22,550 (69.7%)	23,035 (69.7%)
Nonfarmer	218 (31.0%)	95 (29.4%)	82 (33.7%)	9797 (30.3%)	10,015 (30.3%)
Personal annual income (%)					
<30,000	573 (89.0%)	260 (85.5%)	198 (93.8%)	25,622 (89.2%)	26,195 (89.2%)
30,000–80,000	39 (6.0%)	20 (6.6%)	9 (4.3%)	2484 (8.6%)	2523 (8.6%)
>80,000	32 (5.0%)	24 (7.9%)	4 (1.9%)	626 (2.2%)	685 (2.2%)
Smoking (%)					
Never	478 (67.6%)	224 (68.7%)	150 (61.5%)	24,891 (75.4%)	25,369 (75.3%)
Former/current	229 (32.4%)	102 (31.3%)	94 (38.5%)	8101 (24.6%)	8330 (24.7%)
Hypertension (%)					
No	474 (67.2%)	213 (65.3%)	169 (69.3%)	24,726 (74.9%)	25,201 (74.8%)
Yes	232 (32.8%)	113 (34.7%)	75 (30.7%)	8266 (25.1%)	8498 (25.2%)
Diabetes (%)					
No	616 (87.1%)	281 (86.2%)	216 (88.5%)	30,329 (91.9%)	30,945 (91.8%)
Yes	91 (12.9%)	45 (13.8%)	28 (11.5%)	2663 (8.1%)	2754 (8.2%)
IOP (mmHg)^*a*^	17.20 (5.29)	17.77 (6.67)	16.90 (4.87)	14.32 (2.76)	14.38 (2.86)
CACD (CT)^*a*^	2.87 (0.87)	2.41 (0.88)	3.41 (0.91)	3.09 (0.87)	3.09 (0.87)
LACD (CT)^*a*^	0.56 (0.33)	0.43 (0.24)	0.71 (0.37)	0.66 (0.31)	0.66 (0.31)
VCDR^*a*^	0.53 (0.22)	0.59 (0.21)	0.56 (0.22)	0.31 (0.11)	0.31 (0.11)

*PACG* primary angle-closure glaucoma, *POAG* primary open-angle glaucoma, *IOP* intraocular pressure, *CACD* central anterior chamber depth, *LACD* limbal anterior chamber depth, *VCDR* vertical cup-to-disc ratio, *CT* corneal thickness

^*a*^Values were shown as mean (SD)

Of the 33,699 participants, 707 had glaucoma. In those 40 years of age or older, the prevalence of glaucoma was 2.1% (95% CI 1.94–2.25%). The constituent ratios of glaucoma were as follows: PACG, 46.1%; POAG, 34.5%; and other types, 19.4%. The prevalence of glaucoma in men and women was 2.13% and 2.07%, respectively. When compared with those who were married, it was substantially higher among those who had never married (3.25 %; 95% confidence interval (CI): 1.97–4.53%), as well as those who had been divorced or separated (2.88%; 95% CI: 2.23–3.54%). As shown in Table 3, the average IOPs were 17.20 (5.29) mmHg in glaucoma and 14.32 (2.76) mmHg in nonglaucoma. The CACDs were 2.87 (0.87) CT in glaucoma and 3.09 (0.87) CT in nonglaucoma (a corneal thickness of 1 CT).

### Summary of heatwaves, cold spells, temperature, and PM_2.5_

**Fig. 2:**
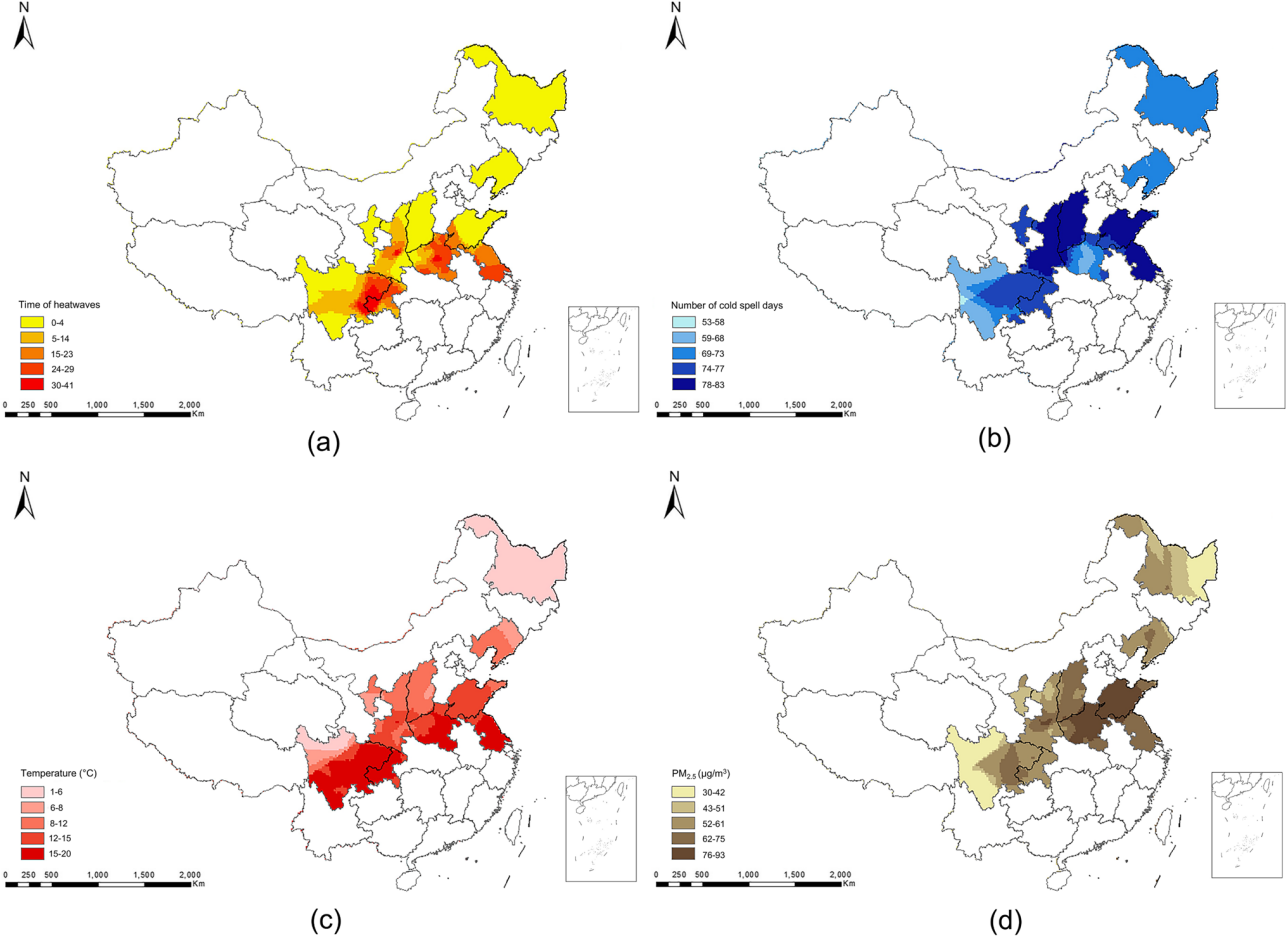
Characteristics of heatwaves, cold spells, temperature, and PM_2.5_ in ten provinces of China from the year of 2007 to 2016. **a** The number of heatwaves from 2007 to 2016. **b** Total days of cold spells from 2007 to 2016. **c** Daily mean temperature from 2007 to 2016. **d** Mean PM_2.5_ exposure by established satellite-based spatiotemporal model. The definitions of heatwaves and cold spells used are HW08 (temperature ≥97th mean temperature and duration ≥ 3 days) and CS04 (5th and 9 days–2 m temperature), respectively. HW: heatwave; CS: cold spell

Characteristics of heatwaves, cold spells, temperature, and PM_2.5_ in ten provinces from 2007 to 2016 are shown in Fig. 2. According to the definitions of heatwaves and cold spells, as shown in Table S1, the heatwave times in Jiangsu, Chongqing, and Henan were slightly more than other provinces. The cold spell days in Shanxi, Jiangsu, and Shaanxi were in the top three. Patients of glaucoma were exposed to more heatwave times than nonglaucoma participants, and glaucoma patients were also exposed to more cold spell days than nonglaucoma participants (Table S2).

Pairwise correlations are exhibited in Fig. S1. The correlation coefficients between PM_2.5_ and heatwaves and PM_2.5_ and temperature were strong. We found a weak and adverse relationship between cold spells and PM_2.5_.

### Associations of heatwaves and cold spells with glaucoma

**Fig. 3:**
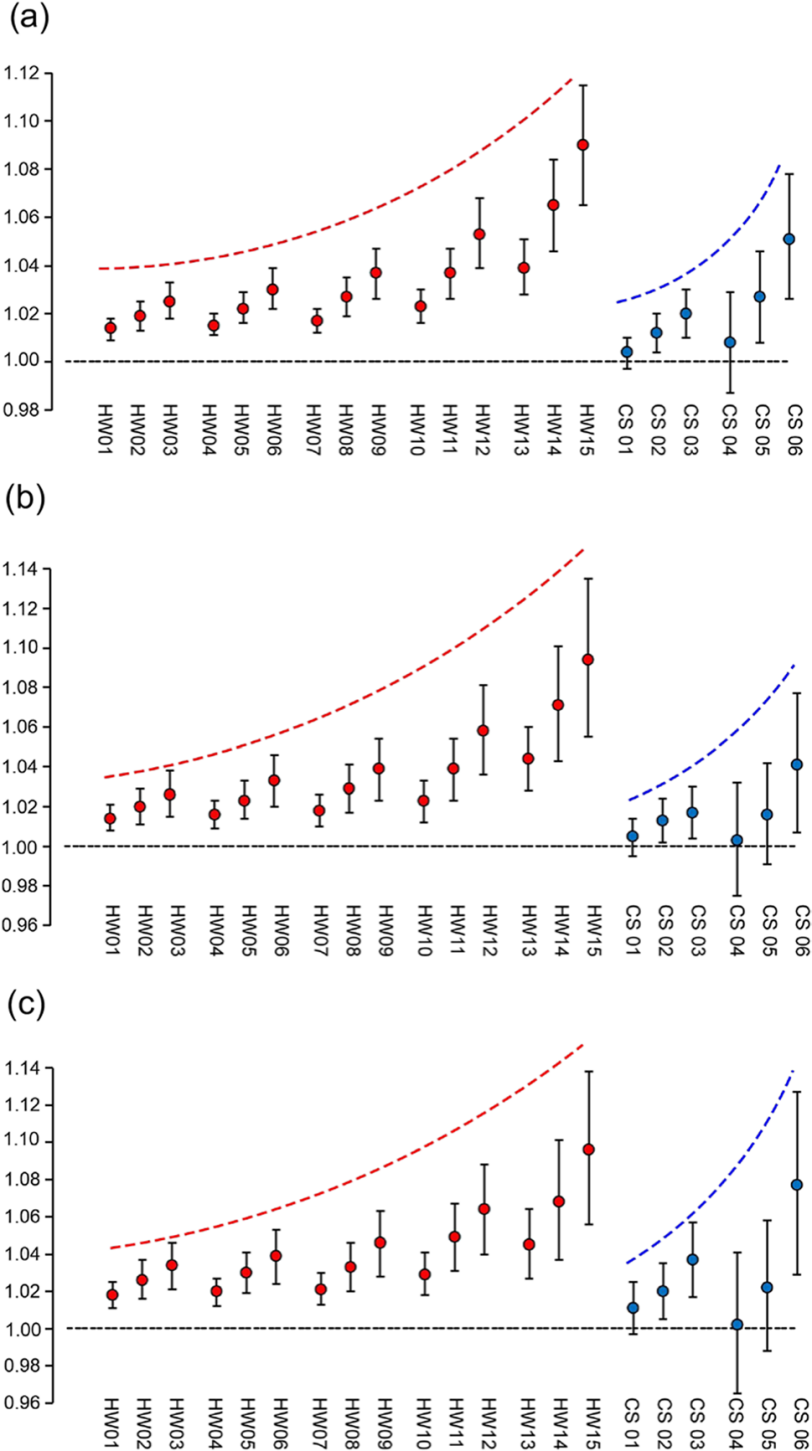
Odds ratios (OR) (95% confidence interval (CI)) for glaucoma correlations with heatwaves and cold spells. **a** OR (95% CI) for correlations with total glaucoma. **b** OR (95% CI) for correlations with primary angle-closure glaucoma (PACG). **c** OR (95% CI) for correlations with primary open-angle glaucoma (POAG). Adjusting for gender, age, regions, education levels, occupation, personal annual income, smoking status, hypertension history, diabetes history, IOP, and PM_2.5_ levels in the model. HW: heatwave; CS: cold spell

Figure 3 and Table S3 show the odds ratio (OR) and 95% CI for associations of heatwaves and cold spells with glaucoma, PACG, and POAG. The OR (95% CI) of glaucoma ranged from 1.014 (1.009, 1.018) for HW01 (temperature ≥95th and duration ≥ 2 days) to 1.090 (1.065, 1.115) for HW15 (temperature ≥99th and duration ≥ 4 days) depending on the variety of heatwave definitions; the OR (95% CI) of PACG ranged from 1.014 (1.008, 1.021) for HW01 to 1.094 (1.055, 1.135) for HW15; and the OR (95% CI) of POAG ranged from 1.018 (1.011, 1.025) for HW01 to 1.096 (1.056, 1.138) for HW15. For cold spells, the OR (95% CI) for glaucoma from CS01 (10th and 9 days–2 m temperature) to CS06 (5th and 3 days–2 m temperature) was 1.004 (0.997, 1.010) to 1.051 (1.026, 1.078); the OR (95% CI) for PACG from CS01 to CS06 was 1.005 (0.995, 1.014) to 1.041 (1.007, 1.077); the OR (95% CI) for POAG from CS01 to CS06 was 1.011 (0.997, 1.025) to 1.077 (1.029, 1.127).

Overall, the risk of glaucoma, PACG, and POAG increased with the intensity of heatwaves and cold spells. As the definition of heatwaves or cold spells becomes more rigorous, a marked increase in OR was observed. With respect to the heatwave effect, the strongest associations were found for glaucoma, PACG, and POAG in HW15, which is 99th and ≥4 days. Heatwaves have stronger effects on glaucoma than cold spells.

The interactions of heatwaves/cold spells with PM_2.5_ were evaluated in multiplicative terms. The interactions of heatwaves/cold spells with PM_2.5_ were evaluated by multiplicative terms. As shown in Table S4, the OR (95% CI) of the interaction term of the cross product between heatwaves and PM_2.5_ were greater than 1 (*P* < 0.05), which suggested heatwaves and PM_2.5_ had a synergistic effect on the prevalence of glaucoma, PACG, and POAG.

### Dose-response relationships of heatwaves/cold spells with IOP, CACD, and glaucoma risk

**Fig. 4:**
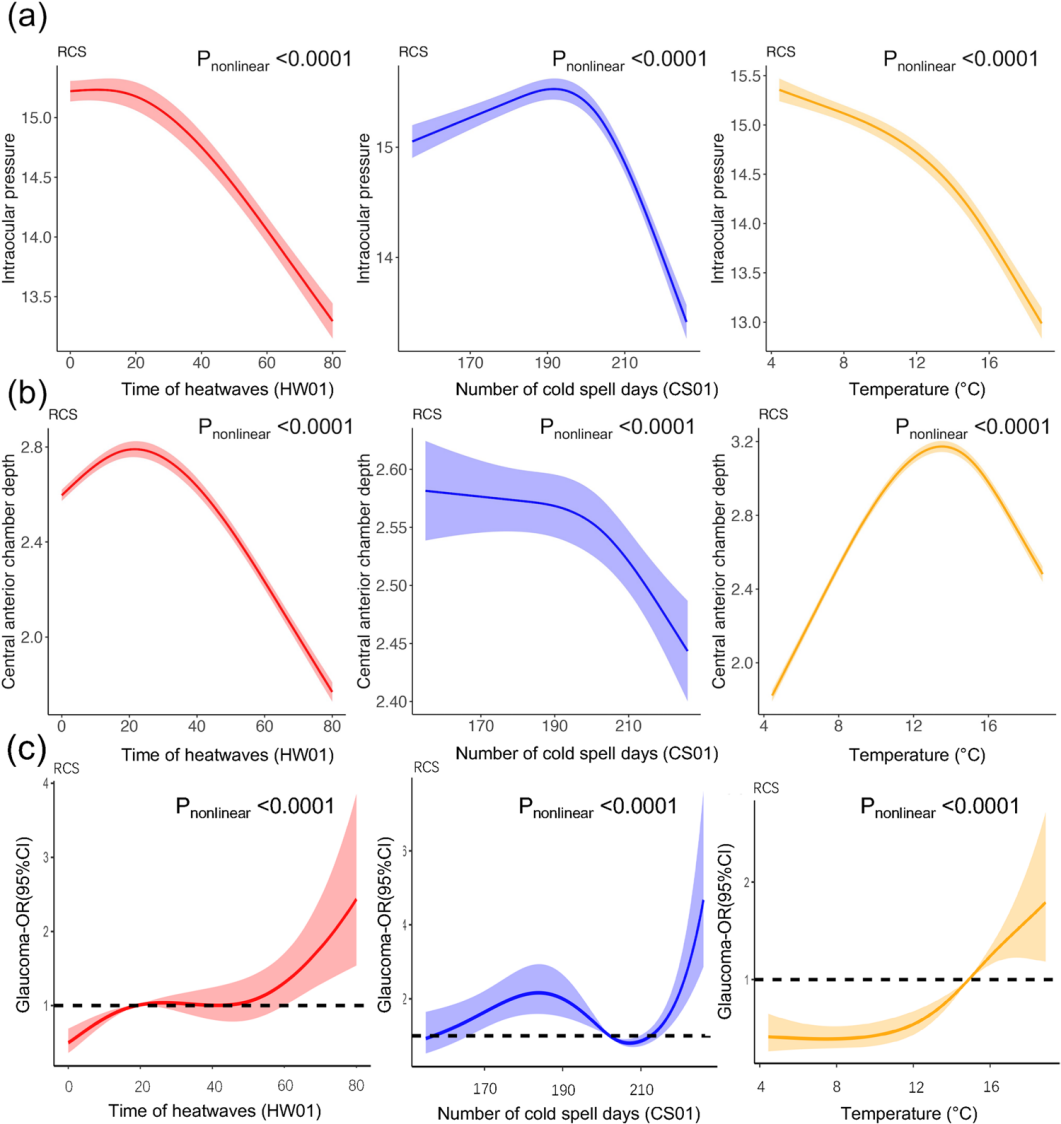
Analysis of dose-response association using RCS function. **a** Adjusting dose-response association between heatwaves, cold spells, and temperature and IOP risk using the RCS function. **b** Adjusting dose-response association between heatwaves, cold spells, and temperature and CACD risk using the RCS function. **c** Adjusting dose-response association between heatwaves, cold spells, and temperature and glaucoma risk using the RCS function. The solid lines represent estimates of the effects, and the dashed line represents the 95%CI. Adjusted for gender, age, region, education, occupation, personal annual income, smoking, hypertension, diabetes, IOP, and PM_2.5_

We utilized RCS in Fig. 4 to model and depict the relationship between heatwaves, cold spells, and temperature with IOP, CACD, and the risk of glaucoma. Nonlinear relationships (nonlinear *P* < 0.001) were observed for heatwaves, cold spells, and temperature with IOP and CACD, with the estimated curves showing a decreasing trend. The relationship between cold spells and IOP showed a weak upward trend when the number of cold spell days was below 198 days and a downward trend when the number of cold spell days exceeded 198 days (Fig. 4a). The relationship between cold spells and CACD shows a moderate trend when the number of cold spell days is below 199 days and a decreasing trend when the number of cold spell days exceeds 199 days. The relationship between temperature and CACD showed an increasing trend when the temperature was less than 14°C and a decreasing trend when it exceeded 14° (Fig. 4b). Heatwaves, cold spells, and temperature and glaucoma risk were nonlinear correlated (nonlinear *P* < 0.001), with the estimated curves showing an increasing trend. Up to 14°, temperature was linked to an OR for glaucoma risk that was less than 1, but after that point, the OR was greater than 1 (Fig. 4c).

### Stratified analysis

**Fig. 5:**
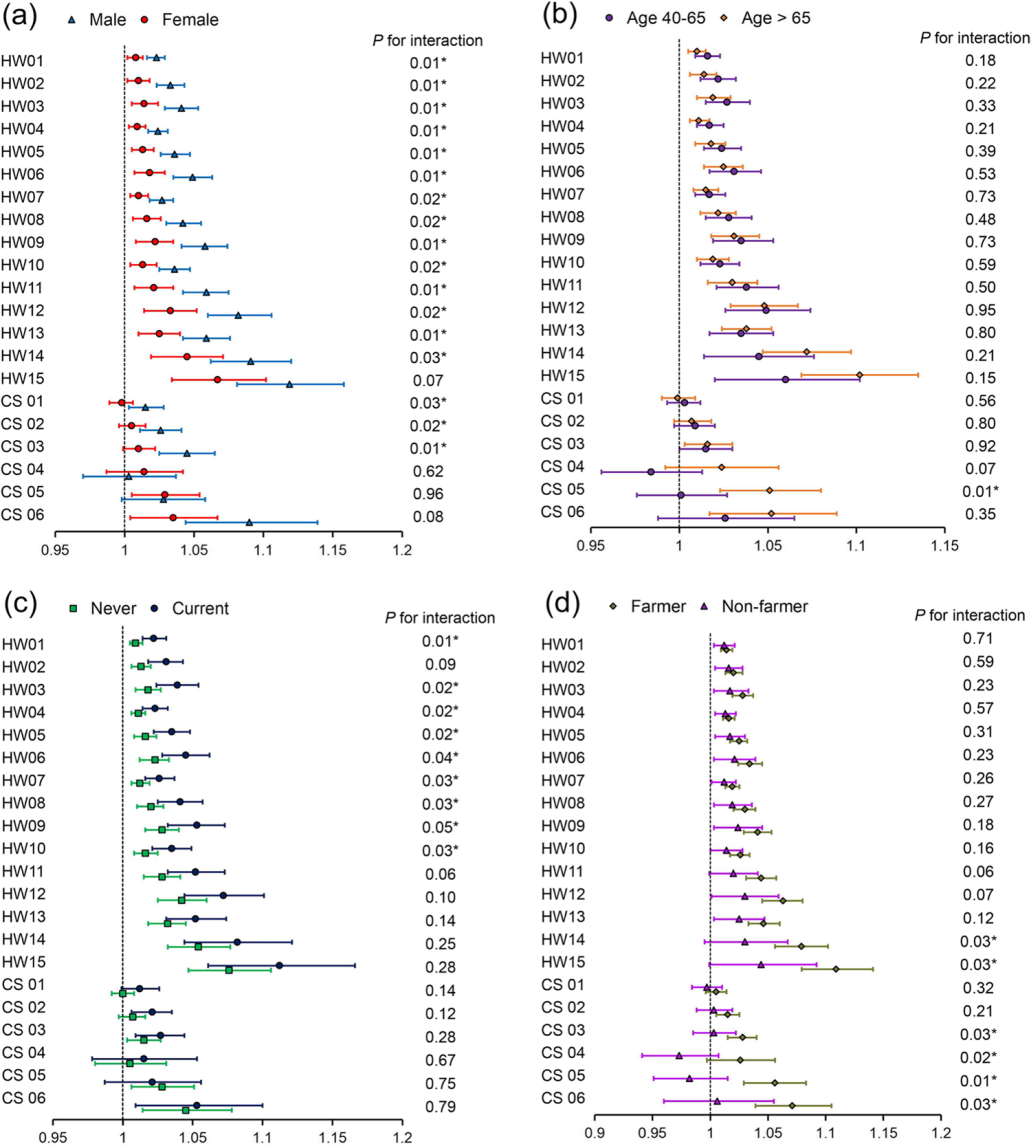
OR (95% CI) for associations of glaucoma with heatwaves and cold spells by stratified analysis. Results from subgroup analyses stratified by sex (**a**), age (**b**), smoking status (**c**), and occupation (**d**) after multivariable adjustments. We observed that the estimated effects (odds ratio) for heatwaves or cold spells on glaucoma were significantly higher in males than in females. For heatwaves with glaucoma, the estimates for the association were significantly larger among current smokers than nonsmokers, farmers than nonfarmers. HW: heatwaves; CS: cold spells. *Represents statistical differences

Subgroup analyses were stratified by gender, age, smoking status, and occupation, as shown in Fig. 5 (glaucoma), Fig. S2 (PACG), and Fig. S3 (POAG). In our subgroup analysis, the estimated effects of heatwaves or cold spells on glaucoma were higher in males than in females. For heatwaves (HW01), the OR was 1.023 (95% CI: 1.016, 1.029) in males and 1.008 (95% CI: 1.002, 1.013) in females. For cold spells (CS01), the OR was 1.015 (95% CI: 1.003, 1.028) in males and 0.998 (95% CI: 0.989, 1.006) in females. If the effects of heatwaves/cold spells on PACG and POAG were analyzed separately, the differences between men and women described above were found in PACG but not in POAG. For heatwaves with glaucoma, the estimates for the association were significantly larger among current smokers (OR = 1.022, 95% CI: 1.014, 1.031 for HW01) than nonsmoking individuals (OR = 1.009, 95% CI: 1.005, 1.014). We found no differences between smokers and nonsmokers that were significant for the impact of cold spells.

As shown in Tables S5, S6, and S7, in the total glaucoma patient and PACG patient populations, we found that those with a family history of glaucoma were more sensitive to cold spells. The OR of CS01 in the study population with a family history of glaucoma was 1.116 (1.031, 1.208), while the OR of CS01 in the study population without a family history of glaucoma was 1.002 (0.995, 1.009), with a statistically significant difference (*P* < 0.05). Although the CI overlapped, the estimate for the association of heatwaves was slightly larger among farmers than nonfarmers. We did not find significant differences among different age groups.

### Sensitivity analysis

The outcomes of the sensitivity analysis matched those of the primary analyses. As shown in Table S8, when we exclude individuals with cataract, the estimated effects on OR of glaucoma were 1.013 (95% CI: 1.008, 1.018) and 1.019 (95% CI: 1.012, 1.026) for HW01 and HW02. As shown in Table S9, no significant change in results was seen when participants with chronic diseases (hypertension and diabetes) were excluded. The results did not change appreciably when we added relative humidity and air pressure in the model (Table S10).

## Discussion

This is the first published study, to our knowledge, to investigate the effects of heatwaves and cold spells on glaucoma, especially in the Chinese population. After adjusting for PM_2.5_ and other covariates, we found that heatwaves and cold spells were both significantly associated with increased glaucoma prevalence, and the risk of glaucoma increased with the intensity of heatwaves and cold spells. The subtype outcomes (PACG and POAG) followed a similar pattern. This study also provided evidence that the estimated effects of heatwaves or cold spells on glaucoma were higher in males than females and in smokers than in nonsmokers.

The prevalence of glaucoma in adults older than 40 years (2.1%) was lower than in earlier epidemiological studies on the disease (He et al. 2015, Pan et al. 2016, Song et al. 2011). PACG was significantly more common. However, the prevalence of POAG was much lower in rural China than in African populations (2.08–7.35%) (Tham et al. 2014), other Asian populations (Bourne et al. 2003, Vijaya et al. 2008), and European populations (1.83–4.06%) (Raychaudhuri et al. 2005). We employed the stricter ISGEO definition, which required optic neuropathy to diagnose glaucoma; this may be a factor in the comparatively lower prevalence of glaucoma in our study. Our results showed that the provinces with the highest number of heatwaves were Jiangsu, Chongqing, and Henan, with the cold spell days in Shanxi, Jiangsu, and Shaanxi being in the top three. This is because, for the relative temperature thresholds used in defining cold spells (5th percentile, 10th percentile), the average daily temperature on cold spell days was higher in warmer regions (i.e., the south) and lower in colder regions (i.e., the north). For example, the annual mean temperature on cold spell days in Jiangsu was 16.13°C but was 4.73°C in Heilongjiang. Due to the lower temperature threshold, the distribution of cold spell days was lower in the northern regions than in most of the southern regions. Our results are in general agreement with another regional distribution of cold spell days in 280 counties in China from 2013 to 2019 (Sun et al. 2022).

This study found that in heatwaves, mean daily temperatures above the 95th percentile (HW15) for more than four consecutive days had the highest risk for glaucoma compared to other definitions; in cold spells, the average 2 m temperature of 3 days in the moving window centered on the study day and daily threshold indices of the 5th (CS06) had the highest risk for glaucoma. However, to date, there is no standard definition of heatwaves and cold spells, mainly because the impact of heatwaves and cold spells depends on many factors, including climate and sociodemographic characteristics (Tong et al. 2015). A further important reason is regional differences in adaptation. The adaptation of populations to the intensity of cold can lead to different health risks at different cold thresholds (Sun et al. 2022).

Overall, in this study, we found that the risk of glaucoma, POAG, and PACG increased with the intensity of the heatwaves and cold spells. Similar findings were observed by a multicenter research conducted in the US comprising 209 cities. Their findings suggest that cold spell-related mortality increased with the duration and intensity of the cold spells (Wang et al. 2016). Two other studies conducted in multiple cities in Australia (Tong et al. 2015) and China (Yang et al. 2019a) exploring the association between heatwaves and mortality found stronger correlations for longer heatwaves with the same temperature threshold as well as for heatwaves defined by higher temperature threshold but the same duration. High ambient temperatures may lead to thermal damage to eye structures as the blood transfers body temperature to the eyes (El Hamichi et al. 2020). Thus, long-term exposure to small increases in temperature may lead to an accelerated aging process in the lens by accelerating the metabolic rate of the lens epithelium (Freeman & Fatt 1973).

In the available literature, data on the association between heatwaves or cold spells and glaucoma are limited. In the UK and Finland, an increased incidence of PACG was reported in the winter, while in Singapore, a higher incidence was found on warmer days (Seah et al. 1997). In a seasonal study of acute episodes of PACG in mainland China, the results showed a higher incidence in the summer and winter (Zhu et al. 2018). A study in Africa suggests that higher ambient temperatures may accelerate the onset of POAG and increase its prevalence (Weale 2007). It has been hypothesized that underlying mechanisms underlie the elevated prevalence of glaucoma linked to extreme temperatures (Liao et al. 2022, Mansouri et al. 2022, Vongsachang et al. 2021, Zhong et al. 2021). Most of the eye’s receptors (70%) are polymodal nociceptors activated by heat, exogenous chemical stimuli, and endogenous chemical agents. A small number of nerves (20%) are mechanical receptors that respond to mechanical forces close to the strength needed to damage corneal epithelial cells. A few (10%) of the cold receptors were silenced when the temperature increased, and the firing rate of the cold receptors was increased when the surface temperature of the cornea decreased (Patel et al. 2021). So sudden physiological responses are consistent with the steep increase in risk that was associated with extreme temperature events (Mansouri et al. 2022, Yang et al. 2019b).

Occluded aqueous humor outflow caused by crowded ocular anatomy or angle closure was linked to an elevation in IOP (Nongpiur et al. 2011). Elevated IOP was regarded as one of the main modifiable risk factors for glaucoma because it can impair optic nerve axon transit (Maddineni et al. 2020). IOP and the optic nerve may be triggered by heatwaves and cold spells via vasoconstriction and inflammatory responses (Baudouin et al. 2021, Khawaja et al. 2014, Shan et al. 2021). As an alternative, temperature stress may influence psychophysiological processes by changing serotonin and dopamine synthesis or interfering with thermoregulation homoeostasis, causing emotional changes such as tension or anxiety (Aghamohammadi et al. 2021, Cianconi et al. 2020, Li et al. 2022, Liu et al. 2021), by affecting the amounts of the hormone cortisol in the systemic circulation, which could raise IOP (Gillmann et al. 2019, Ikegami et al. 2020, Zhong et al. 2021). The estimated curves showed a decreasing trend, considering the observed nonlinear relationship between heatwaves, cold spells, and temperature and IOP. It shows that the elevated likelihood of glaucoma linked with heatwaves and cold spells may be caused by biological processes other than the IOP raising pathway.

Shallow anterior chamber depth (ACD) can lead to obstruction of atrial fluid drainage and reverse flow into the vitreous humor. The shallower the ACD, the greater the chance of atrial angle closure and the higher the incidence of PACG (Nongpiur et al. 2011). Therefore, measurement of the anterior chamber is an important sign for acute closed-angle glaucoma, especially the depth of the CACD, to determine whether there is a risk of closed-angle glaucoma (Wang et al. 2019). The dose-response relationship curve showed a decreasing trend in the nonlinear relationship between cold spells and CACD. At temperatures below 14 °C, the CACD became shallower as the temperature decreased. Studies have shown that the shallow anterior chamber had a thermoregulatory effect and that a shallow CACD brought the iris closer to the cornea to resist corneal freezing, thus making the eye more adaptable to cold climates (Casson 2008). It was found that Greenlandic Inuit have a typical shallow anterior chamber and reported a higher incidence of PACG (Alsbirk 1992, Drance 1973, Wojciechowski et al. 2003).

Our results identified a higher risk for men compared with women, which was different from some other studies. Some studies suggested that most women have more difficulty regulating their internal temperature (Kollanus et al. 2021, Laaidi et al. 2006, Moraes et al. 2022). According to some research, men were more susceptible to excessive temperatures, which may be related to the larger percentage of male daily wage earners in the working-age population (Kumar &Singh 2021). A stronger effect in this study could be partly explained by the fact that men spend more time outdoors in rural areas. Hence, in this rural population, men had higher effects than women. Our results indicated that smokers were confirmed to have higher susceptibility to heatwaves and cold spells. The adaptation to extreme temperatures may contribute to the susceptibility of smokers. One possible mechanism is that smokers have a weaker vascular circulation system, which affects IOP in the long term (Xu et al. 2021). These theories and mechanisms need to be confirmed by additional research.

Our study has several limitations. First, this study is based on the data of a cross-sectional study. A prospective study on extreme climate and glaucoma needs to be conducted in the future. Second, the average daily temperature is not equivalent to the temperature averaged over the entire day. No more precise temperature data were available for analysis. Third, this study focused on the effects of heatwaves and cold spells on glaucoma, and did not consider indoor temperature regulation. Future studies may include the use of personal monitors to examine real-time personal-level temperature changes and the addition of questions to questionnaires to determine the effects of factors such as participants’ indoor stay times. Additionally, only one kind of air pollution, PM_2.5_, was included, and the role of air pollution in the effects of heat and cold on glaucoma was not elucidated.

## Conclusions

We examined the impacts of heatwaves and cold spells on glaucoma based on the REG-China study. Both heatwaves and cold spells were positively related to glaucoma prevalence. The OR for glaucoma increased with the intensity of heatwaves and cold spells. There is a need for further research on the impact of extreme weather events on the incidence of and access to eye diseases for specific causes, socioeconomic conditions, and climate types, with public health implications for local communities.

## Supplementary information

ESM 1

## Data Availability

The datasets used and/or analyzed during the current study are available from the corresponding author on reasonable request.

## Abbreviations

AOD: Aerosol optical depth
ACD: Anterior chamber depth
CACD: Central anterior chamber depth
CG: Congenital glaucoma
CS: Cold spell
CI: Confidence interval
ERA5: European Centre for Medium-Range Weather Forecasts reanalysis v5
EWEs: Extreme weather events
HW: Heatwave
IOP: Intraocular pressure
ISGEO: International Society of Geographical and Epidemiological Ophthalmology
MAIAC: Multi-Angle Implementation of Atmospheric Correction
MODIS: Moderate Resolution Imaging Spectrometer
NASA: National Aeronautics and Space Administration
OR: Odds ratio
POAG: Primary open-angle glaucoma
PACG: Primary angle-closure glaucoma
RCS: Restricted cubic spline
REG-China: Glaucoma Rural Epidemiology in China
