# Supplementary material for: Impacts of heatwaves and cold spells on glaucoma in rural China: a national cross-sectional study
Source: Environ Sci Pollut Res Int. 2023 Feb 3;30(16):47248–61. doi: 10.1007/s11356-023-25591-8 (PMC10097786; doi:10.1007/s11356-023-25591-8)
Supplement: Supplementary file 1 — ESM 1 [file 11356_2023_25591_MOESM1_ESM.docx]

**Supplemental Materials**

**Title**

Impacts of heatwaves and cold spells on glaucoma in rural China: a national cross-sectional study.

**Journal name**

Environmental Science and Pollution Research

**Authors**

Ai Zhang, Qihua Wang, Xueli Yang, Yuanyuan Liu, Jiayu He, Anqi Shan, Naixiu Sun, Qianfeng Liu, Baoqun Yao, Fengchao Liang, Ze Yang, Xiaochang Yan, Shaoye Bo, Yang Liu, Hongjun Mao, Xi Chen, Nai-jun Tang, Hua Yan

**Corresponding authors**

Hua Yan, MD, PhD.

Department of Ophthalmology, Tianjin Medical University General Hospital, Laboratory of Molecular Ophthalmology and Tianjin Key Laboratory of Ocular Trauma, Tianjin Medical University, No. 154, Anshan Road, Tianjin, China 300052

Fax: 86-22-27813550

Email: [zyyyanhua@tmu.edu.cn](mailto:zyyyanhua@tmu.edu.cn)

**Table of Contents**

**Part 1: Supplemental Methods**

**Part 2: Supplemental Figures**

**Fig.S1.** Spearman correlation of temperature, cold spells, heatwaves and PM_2.5_.

**Fig.S2.** OR (95% CI) for associations of PACG with heatwaves and cold spells by subgroups.

**Fig.S3.** OR (95% CI) for associations of POAG with heatwaves and cold spells by subgroups.

**Part 3: Supplemental Tables**

**Table S1:** Summary of temperature, heatwaves, cold spells and Glaucoma in ten provinces of China.

**Table S2:** Exposure of heatwaves and cold spells in Glaucoma and Non-glaucoma participants.

**Table S3:** OR (95% CI) for associations of glaucoma with heatwaves and cold spells.

**Table S4:** Adjusted OR (95%CI) for the multiplicative interaction of heatwaves, cold spells and PM_2.5_.

**Table S5:** According to the subgroup of glaucoma family history, the OR (95% CI) for the association of glaucoma with heatwaves and cold spells.

**Table S6:** According to the subgroup of glaucoma family history, the OR (95% CI) for the association of PACG with heatwaves and cold spells.

**Table S7:** According to the subgroup of glaucoma family history, the OR (95% CI) for the association of POAG with heatwaves and cold spells.

**Table S8:** Sensitivity analysis for exclusion of 3927 cataract patients.

**Table S9.** Sensitivity analysis for exclusion of 8498 hypertension patients and 2754 diabetes patients.

**Table S10:** Sensitivity analysis for adjusting relative humidity and air pressure.

**Part 1: Supplemental Methods**

**Study population**

We used a multistage stratified cluster sampling procedure to enroll a nationally representative sample population, aged 6 years or older. A flowchart of the study’s disposition is presented in **Fig.1**. The study protocol was approved by the Ethics Review Committee of Tianjin Medical University and other participating institutes. Written informed consent was obtained from all participants. Shandong and Jiangsu Provinces were sampled from East China, Shanxi Province was sampled from North China, Heilongjiang and Liaoning Provinces were sampled from Northeast China, Ningxia Hui Autonomous Region, and Shaanxi Province were sampled from Northwest China, Henan Province was sampled from South Central China, and Sichuan Province and Chongqing Municipality were sampled from Southwest China. We used a multistage stratified cluster sampling procedure to enroll a nationally representative sample of populations. It was stratified by region and province, and counties and townships were further selected for each province. The population aged 6 years and above in the sampled districts was investigated and screened. All participants were contacted telephonically and agreed to visit the local community hospital for examination. All organizations and contacts were examined with the assistance of the local community (neighborhood committee) staff. The local community staff understood the household registration and residence information of the community and more frequently came in contact with residents in their daily work. The organization and contact of community workers can enhance the compliance of the population. Standardized training was conducted for all participating clinicians in the early stages of project implementation, and training manuals were developed. The calibration of inspection instruments in each area was also standardized.

**Questionnaires and Eye Examination**

Detailed interviewer-administered questionnaires were used to collect demographic information, medical history, history of ocular diseases, and family history of glaucoma.

Each participant underwent a standard ophthalmological examination, including distance visual acuity (VA) testing, best-corrected visual acuity (BCVA), slit-lamp microscopy (particularly for limbal anterior chamber depth [LACD]), direct ophthalmoscopy, and slit-lamp biomicroscopy with a 90D convex lens (particularly for vertical cup-to-disc ratio [VCDR]) without pupil dilation, and measurement of intraocular pressure (IOP) with non-contact tonometry.

Participants with any of the following signs were classified as glaucoma suspects according to Foster’s study:^1^ VCDR or VCDR asymmetry ≥ 97.5th percentile (VCDR ≥ 0.6 in either eye or VCDR asymmetry ≥ 0.2 in our study), neuroretinal rim width ≤ 0.1 CDR (between the 11 and 1 o’clock or 5 and 7 o’clock positions), optic disc hemorrhage, notch, nerve-fiber-layer defect; IOP > 97.5th percentile (21 mmHg in our study) in either eye; and LACD ≤ 40% of corneal thickness in either eye. Glaucoma suspects underwent further investigations, including fundus photography, Goldmann applanation tonometry, visual field, and gonioscopy.^2^

Distance VA was assessed monocularly using a 5-m standard logarithmic VA E chart (Tianjin ZhengDa Medical Care). Subjective refraction was performed on all participants with vision worse than 1.0 (less than 20/20) in either eye with an Autorefractor-Keratometer (KR8100, Topcon), and refinement of the sphere, cylinder, and cylinder axis were performed until BCVA was obtained by the trained optometrists.

Slit-lamp microscopy was conducted by trained ophthalmologists to identify LACD (graded as a percentage fraction of the thickness of the adjacent cornea in seven categories: 0%, 5%, 15%, 25%, 40%, 75%, and ≥100%, referenced on standard photographs) and abnormal anterior segment according to Foster’s study.^2^

Fundus examination included the optic disk, retina, vessels, and macula. In particular, the VCDR (referenced on standard photographs) was measured using direct ophthalmoscopy and slit-lamp biomicroscopy with a 90D convex lens for all participants without pupil dilation by a specific glaucoma expert in each province. Some of the participants were randomly selected for examination by our glaucoma expert. Dr. Yao again in each rural village to ensure the consistency of the VCDR estimation. The maximum inflection of the vessels crossing the neuroretinal rim defines the cup margins of the VCDR. Fundus photography (45° of the field centered on the optic disc and macula) was captured. If no clear fundus photography was obtained, the pupil was dilated, provided that the participant did not have closed angles. In the participants with small pupils because of posterior synechia of the iris or unclear refractive media despite pharmacological dilation, a “vague fundus” was recorded.

Non-contact tonometry (NIDEK NT-2000) was performed by experienced technicians and repeated thrice with the mean value recorded. Goldmann applanation tonometry (Tonometer AT/Q900 M ) was performed by experienced ophthalmologists for glaucoma suspects under topical anesthesia, which was considered the final IOP measurement. Two measurements were collected for each eye with the mean value recorded, and a third measurement was performed if the difference between the preceding two measurements was above 2 mmHg.

White-on-white automated perimetry was conducted by experienced technicians for glaucoma suspects after near-refractive correction (Humphrey Field Analyzer 750i). Visual field testing was repeated if test reliability was unsatisfactory (fixation loss >20%, false-positive rate >33%, or false-negative rate >33%). For the visual field defect as per the International Society for Geographical and Epidemiological Ophthalmology (ISGEO), the glaucoma hemifield test was graded “outside normal limits” and a cluster of three contiguous points at the 5% level on the pattern deviation plot consistent with the optic structural changes was selected” based on the study published by Paul Foster.^1^

Static gonioscopy was performed on glaucoma suspects with a Goldmann-type one-mirror lens under dim ambient illumination by experienced ophthalmologists. A dynamic examination with increased illumination and slit height was performed. For participants in whom the iridotrabecular contact could not be satisfactorily reversed, a four-mirror lens was used, and gentle pressure was placed on the cornea. The angle graded using Scheie grading system was documented.^3^

**Quality Control**

The ophthalmologists performing the above contents were trained before the study, and repeatability tests were performed using the weighted kappa method. The consistency rates of LACD, VCDR, IOP, and gonioscopy examinations were 78.6%, 79.8–85.3%, 79.2–84.7%, and 76.4%, respectively. For each province, there were approximately 5–7 ophthalmologists: one glaucoma specialist, one glaucoma fellow, three to five residents, and one technician who was trained to participate in the study. Before the study started, the training consisted of three parts as followed. First, the China Rural Glaucoma Prevention Project expert group trained all ophthalmologists and technicians on filling in the screening questionnaire and diagnosis of glaucoma in Tianjin. Second, Dr. Yao (glaucoma specialist, co-author of this manuscript) went to each study site to train the ophthalmologists (glaucoma fellow, residents) and technicians who attended the study scene regarding the screening questionnaire and ophthalmic testing. Finally, a 3-day pilot study (approximately 1000 people) was performed before the real study, which primarily tested the consistency and reliability of the inspection results of several teams through the implementation of a complete test process in a relatively large sample. After the technical advisory committee approved the work of the pilot study, on-site screening was formally conducted. To ensure quality control, all ophthalmologists and technicians have to accomplish the above training plan, pass the examinations and certified as members of the study team.

**Case Definition**

Glaucoma was categorized into three categories according to the ISGEO criteria.^1,4^ Primary open-angle glaucoma (POAG) was defined as an eye with evidence of glaucoma (optic nerve damage meeting any of the three categories of the evidence above); however, no evidence of angle-closure on gonioscopy and no identifiable secondary cause. Primary angle-closure suspects (PACS), primary angle-closure (PAC), and primary angle-closure glaucoma (PACG) were diagnosed according to the Association of International Glaucoma Societies, in combination with a history of an acute attack of symptomatic elevation of IOP and remaining signs after acute angle-closure, or chronic IOP elevation, or eye signs of filtering surgery, peripheral iridectomy, or laser peripheral iridectomy. For participants with bilateral pseudophakia and/or aphakic eyes, documented records were considered suggestive; otherwise, open-angle glaucoma was recorded as diagnosis.^5^ Congenital glaucoma (CG) included primary and secondary forms of childhood glaucoma.^6,7^ Other types of glaucoma related to uveitis, trauma, corticosteroids, intraocular neoplasms, rubeosis, intraocular surgeries, or ambiguous causes met the criteria for categories 1–3 were included.

Blindness was defined as BCVA in the better-seeing eye of <20/400 according to the World Health Organization criteria.

**Exposure assessment for PM_2.5_**

The two-step process of filling the AOD missingness in the personal PM_2.5_ exposure assessment. First, a daily simple linear regression was performed to impute the missingness for AOD. The regression coefficients were used to estimate the missing Aqua or Terra AOD when only one of them was present,^8^ and then daily average AOD in each grid cell was used. Second, multiple imputation with an additive model was performed to fill in the remaining missingness, and the predictors included MODIS cloud fraction, humidity in the boundary layer, temperature, elevation, albedo, total column water, and the AOD from modern-era retrospective analysis for research and applications.^9^

**Part 2: Supplemental Figures**


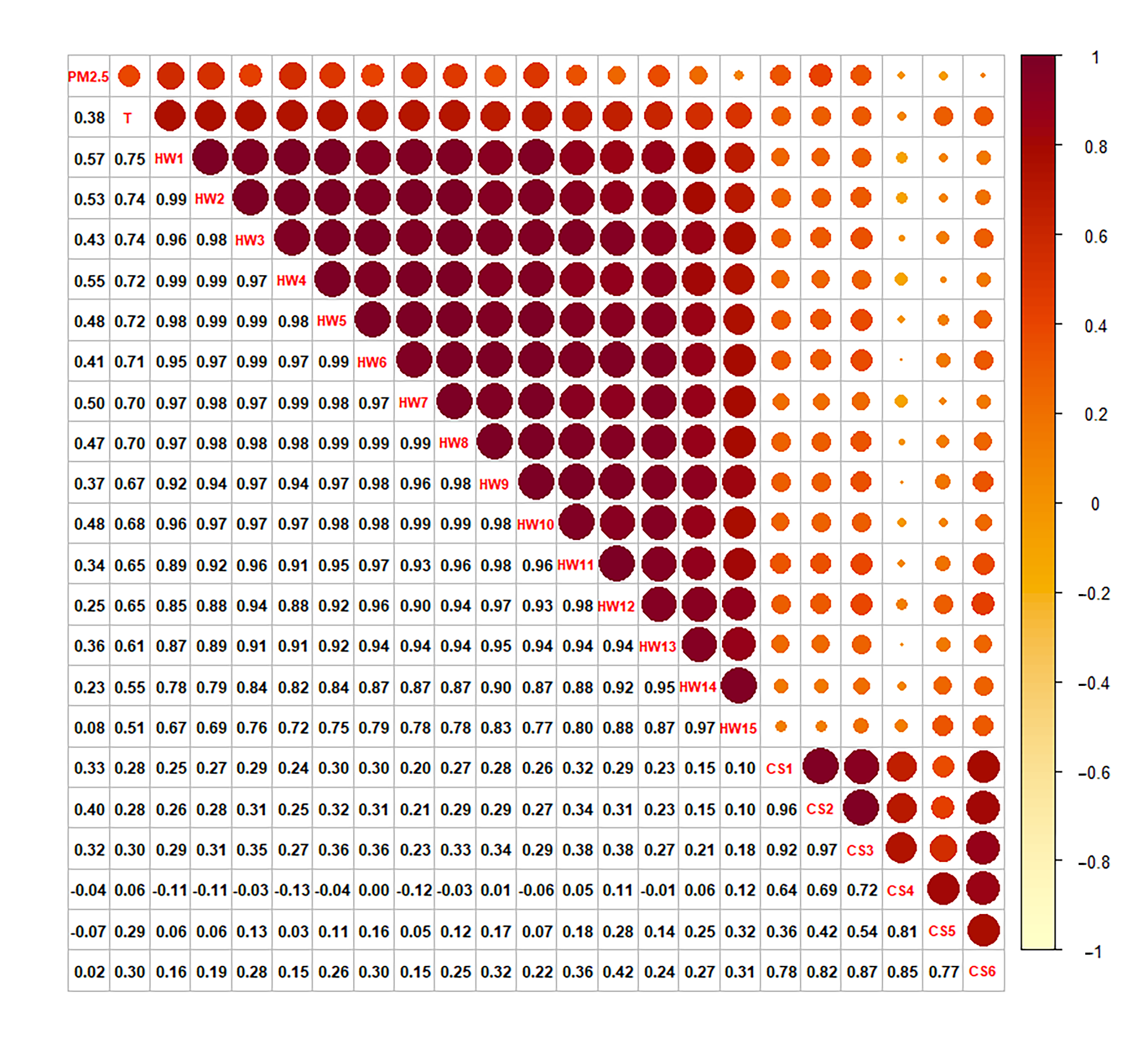


**Fig.S1** Spearman correlation of temperature, cold spells, heatwaves and PM_2.5_. The highest correlation is 1 for positive correlation (red), the lowest correlation is -1 for negative correlation (yellow), and the size of the dots represents the correlation coefficient value

**
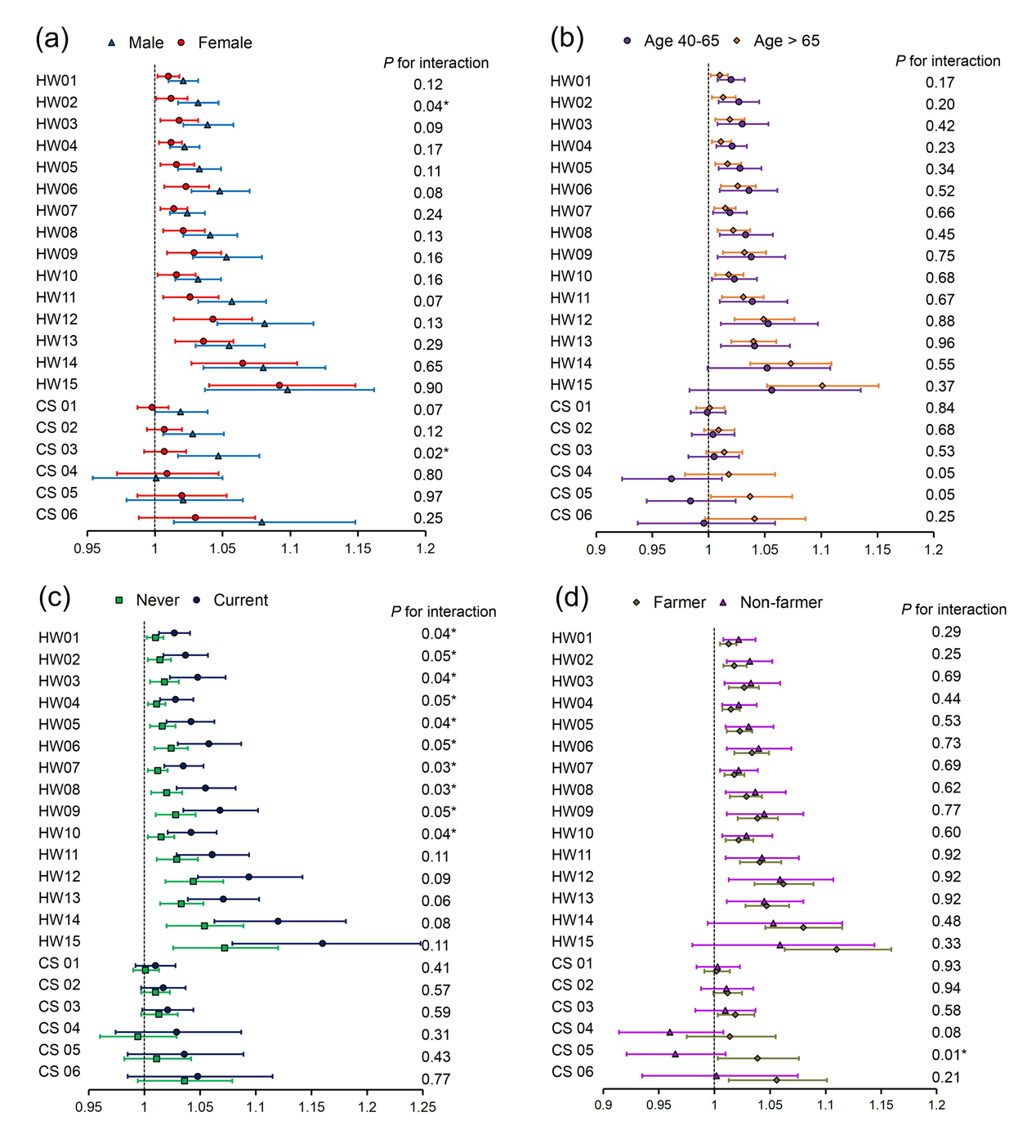
**

**Fig.S2** OR (95% CI) for associations of PACG with heatwaves and cold spells by subgroups. Results from subgroup analyses stratified by sex, age, smoking and occupation after multivariable adjustments


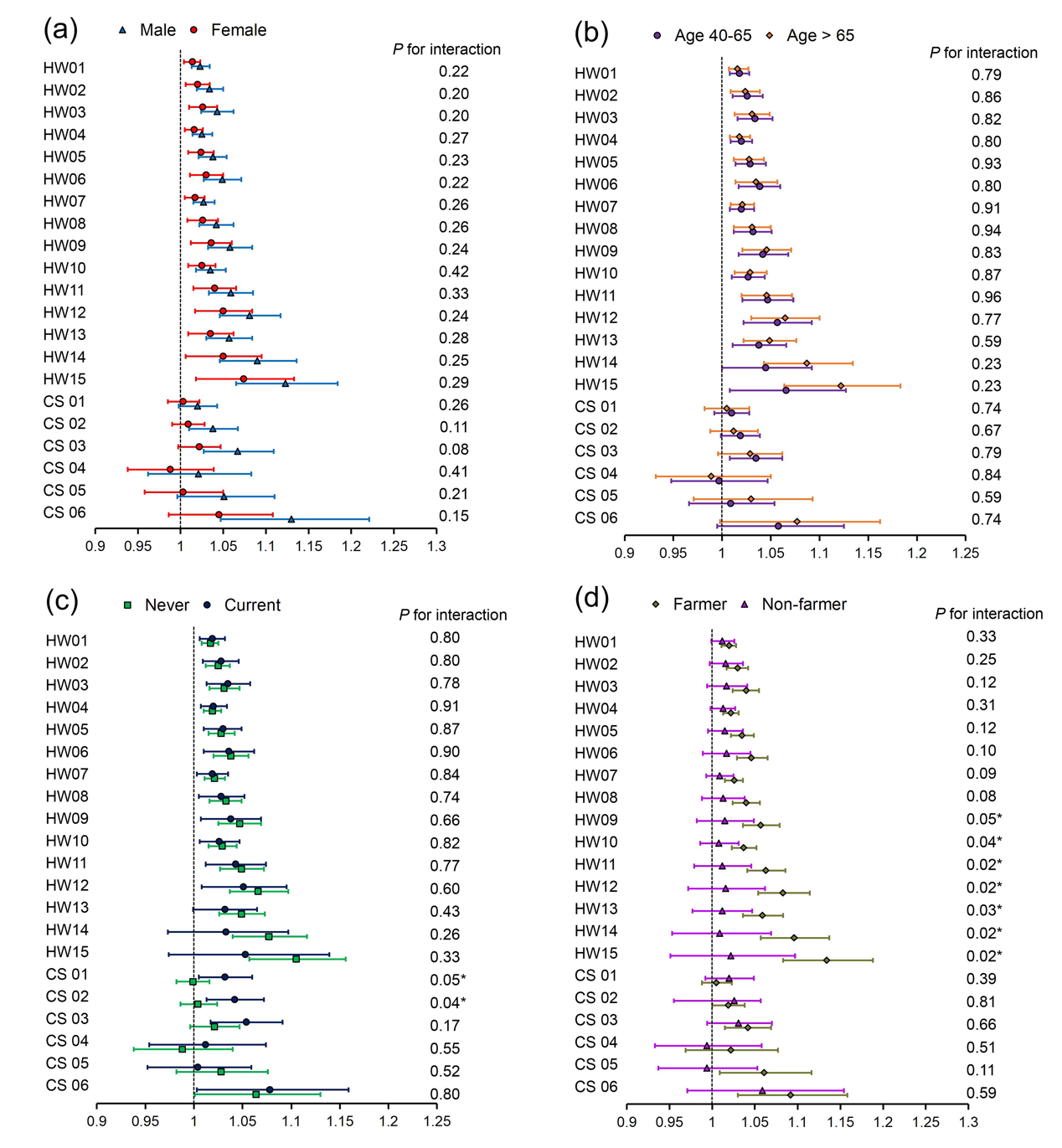


**Fig.S3** OR (95% CI) for associations of POAG with heatwaves and cold spells by subgroups. Results from subgroup analyses stratified by sex, age, smoking and occupation after multivariable adjustments

**Part 3: Supplemental Tables**

**Table S1.** Summary of temperature, heatwaves, cold spells, and Glaucoma in ten provinces of China.

|  | Temperature | | HW08 | | CS04 | | Participants (N) | Glaucoma (N) | PACG (N) | POAG (N) |
| --- | --- | --- | --- | --- | --- | --- | --- | --- | --- | --- |
|  | Mean (℃) | Range (℃) *^a^* | Mean (times) | Range (times) | Mean (days) | Range (days) |  |  |  |  |
| Shandong | 14.04 | 12.16-15.19 | 12.65 | 0-23 | 78.39 | 73-82 | 4310 | 63 | 37 | 13 |
| Jiangsu | 16.13 | 15.82-16.40 | 24.09 | 21-28 | 80.50 | 79-82 | 5092 | 167 | 86 | 70 |
| Ningxia | 7.19 | 6.25-10.10 | 0.00 | 0-0 | 76.07 | 68-80 | 3536 | 30 | 5 | 15 |
| Shaanxi | 13.21 | 10.19-16.01 | 12.30 | 0-38 | 80.47 | 75-83 | 3119 | 39 | 18 | 14 |
| Sichuan | 16.07 | 10.18-18.99 | 3.12 | 0-37 | 73.22 | 10-77 | 3841 | 116 | 45 | 48 |
| Chongqing | 17.47 | 14.56-19.00 | 21.21 | 0-44 | 73.82 | 71-76 | 4052 | 102 | 21 | 49 |
| Shanxi | 10.45 | 7.82-14.53 | 0.01 | 0-12 | 80.81 | 75-83 | 2829 | 33 | 14 | 6 |
| Heilongjiang | 4.73 | 4.44-4.93 | 0.62 | 0-1 | 70.93 | 65-72 | 2904 | 68 | 46 | 5 |
| Liaoning | 9.53 | 3.33-10.97 | 0.00 | 0-1 | 69.21 | 52-83 | 1026 | 13 | 5 | 8 |
| Henan | 15.27 | 11.00-16.60 | 21.45 | 0-38 | 67.40 | 63-82 | 2990 | 76 | 49 | 16 |
| Total | 13.07 | 3.33-19.00 | 11.26 | 0-44 | 75.82 | 10-83 | 33699 | 707 | 326 | 244 |

*^a^* indicates the range of mean temperature for the decade 2007-2016.

**Table S2.** Exposure of heatwaves and cold spells in Glaucoma and Non-glaucoma participants.

|  | Glaucoma | | Non-glaucoma | | Total | | P-value for two groups |
| --- | --- | --- | --- | --- | --- | --- | --- |
|  | mean | range | mean | range | mean | range |  |
| HW01 | 35.05 | 0-80 | 28.46 | 0-82 | 28.6 | 0-82 | P < 0.05 |
| HW02 | 22.18 | 0-54 | 17.79 | 0-59 | 17.88 | 0-59 | P < 0.05 |
| HW03 | 15.75 | 0-37 | 12.29 | 0-43 | 12.36 | 0-43 | P < 0.05 |
| HW04 | 28.95 | 0-67 | 23.11 | 0-78 | 23.24 | 0-78 | P < 0.05 |
| HW05 | 19.10 | 0-46 | 14.98 | 0-50 | 15.07 | 0-50 | P < 0.05 |
| HW06 | 13.05 | 0-34 | 10.04 | 0-39 | 10.10 | 0-39 | P < 0.05 |
| HW07 | 21.79 | 0-62 | 17.32 | 0-64 | 17.42 | 0-64 | P < 0.05 |
| HW08 | 14.29 | 0-39 | 11.19 | 0-44 | 11.26 | 0-44 | P < 0.05 |
| HW09 | 9.85 | 0-32 | 7.50 | 0-32 | 7.55 | 0-32 | P < 0.05 |
| HW10 | 15.37 | 0-49 | 12.10 | 0-56 | 12.16 | 0-56 | P < 0.05 |
| HW11 | 9.25 | 0-30 | 6.94 | 0-32 | 6.99 | 0-32 | P < 0.05 |
| HW12 | 6.40 | 0-24 | 4.73 | 0-28 | 4.76 | 0-28 | P < 0.05 |
| HW13 | 7.93 | 0-31 | 5.90 | 0-36 | 5.95 | 0-36 | P < 0.05 |
| HW14 | 3.76 | 0-19 | 2.82 | 0-24 | 2.84 | 0-24 | P < 0.05 |
| HW15 | 2.49 | 0-15 | 1.78 | 0-19 | 1.80 | 0-19 | P < 0.05 |
| CS01 | 199.30 | 163-226 | 199.73 | 98-226 | 199.72 | 98-226 | P = 0.39 |
| CS02 | 187.93 | 153-214 | 187.60 | 78-214 | 187.60 | 78-214 | P < 0.05 |
| CS03 | 170.81 | 143-199 | 170.04 | 76-199 | 170.22 | 76-199 | P = 0.09 |
| CS04 | 75.40 | 65-83 | 75.83 | 10-83 | 75.82 | 10-83 | P < 0.05 |
| CS05 | 65.20 | 49-76 | 64.89 | 15-76 | 64.90 | 15-76 | P < 0.05 |
| CS06 | 47.26 | 38-57 | 46.98 | 13-57 | 46.98 | 13-57 | P = 0.98 |

Wilcoxon rank sum test was used for testing differences between the two groups.

**Table S3.** OR (95% CI) for associations of glaucoma, PACG, and POAG with heatwaves and cold spells.

|  | Glaucoma | PACG | POAG |
| --- | --- | --- | --- |
| HW01 | 1.014 (1.009,1.018) | 1.014 (1.008,1.021) | 1.018 (1.011,1.025) |
| HW02 | 1.019 (1.013,1.025) | 1.020 (1.011,1.029) | 1.026 (1.016,1.037) |
| HW03 | 1.025 (1.018,1.033) | 1.026 (1.015,1.038) | 1.034 (1.021,1.046) |
| HW04 | 1.015 (1.011,1.020) | 1.016 (1.009,1.023) | 1.020 (1.012,1.027) |
| HW05 | 1.022 (1.016,1.029) | 1.023 (1.014,1.033) | 1.030 (1.019,1.041) |
| HW06 | 1.030 (1.022,1.039) | 1.033 (1.020,1.046) | 1.039 (1.024,1.053) |
| HW07 | 1.017 (1.012,1.022) | 1.018 (1.010,1.026) | 1.021 (1.013,1.030) |
| HW08 | 1.027 (1.019,1.035) | 1.029 (1.017,1.041) | 1.033 (1.020,1.046) |
| HW09 | 1.037 (1.026,1.047) | 1.039 (1.023,1.054) | 1.046 (1.028,1.063) |
| HW10 | 1.023 (1.016,1.030) | 1.023 (1.012,1.033) | 1.029 (1.018,1.041) |
| HW11 | 1.037 (1.026,1.047) | 1.039 (1.023,1.054) | 1.049 (1.031,1.067) |
| HW12 | 1.053 (1.039,1.068) | 1.058 (1.036,1.081) | 1.064 (1.040,1.088) |
| HW13 | 1.039 (1.028,1.051) | 1.044 (1.028,1.060) | 1.045 (1.027,1.064) |
| HW14 | 1.065 (1.046,1.084) | 1.071 (1.043,1.101) | 1.068 (1.037,1.101) |
| HW15 | 1.090 (1.065,1.115) | 1.094 (1.055,1.135) | 1.096 (1.056,1.138) |
| CS01 | 1.004 (0.997,1.010) | 1.005 (0.995,1.014) | 1.011 (0.997,1.025) |
| CS02 | 1.012 (1.004,1.020) | 1.013 (1.002,1.024) | 1.020 (1.005,1.035) |
| CS03 | 1.020 (1.010,1.030) | 1.017 (1.004,1.030) | 1.037 (1.017,1.057) |
| CS04 | 1.008 (0.987,1.029) | 1.003 (0.975,1.032) | 1.002 (0.965,1.041) |
| CS05 | 1.027 (1.008,1.046) | 1.016 (0.991,1.042) | 1.022 (0.988,1.058) |
| CS06 | 1.051 (1.026,1.078) | 1.041 (1.007,1.077) | 1.077 (1.029,1.127) |

Adjusted for gender, age, region, education, occupation, personal annual income, smoking, hypertension, diabetes, IOP, and PM_2.5_.

**Table S4.** Adjusted OR (95%CI) for the multiplicative interaction of heatwaves, cold spells and PM_2.5_.

|  | Glaucoma | PACG | POAG |
| --- | --- | --- | --- |
| HW01×PM_2.5_ | 1.001 (1.000,1.001) * | 1.001 (1.000,1.002) * | 1.001 (1.000,1.002) * |
| HW02×PM_2.5_ | 1.001 (1.001,1.002) * | 1.002 (1.001,1.003) * | 1.002 (1.001,1.003) * |
| HW03×PM_2.5_ | 1.003 (1.002,1.004) * | 1.003 (1.002,1.005) * | 1.004 (1.002,1.005) * |
| HW04×PM_2.5_ | 1.001 (1.001,.1002) * | 1.001 (1.001,1.002) * | 1.001 (1.001,1.002) * |
| HW05×PM_2.5_ | 1.002 (1.001,1.003) * | 1.002 (1.001,1.004) * | 1.003 (1.001,1.004) * |
| HW06×PM_2.5_ | 1.003 (1.002,1.004) * | 1.004 (1.002,1.006) * | 1.004 (1.002,1.006) * |
| HW07×PM_2.5_ | 1.001 (1.001,1.002) * | 1.002 (1.001,1.003) * | 1.002 (1.001,1.003) * |
| HW08×PM_2.5_ | 1.003 (1.002,1.004) * | 1.003 (1.002,1.005) * | 1.003 (1.001,1.005) * |
| HW09×PM_2.5_ | 1.004 (1.003,1.006) * | 1.005 (1.003,1.007) * | 1.005 (1.003,1.008) * |
| HW10×PM_2.5_ | 1.002 (1.001,1.003) * | 1.002 (1.001,1.004) * | 1.003 (1.001,1.004) * |
| HW11×PM_2.5_ | 1.005 (1.003,1.006) * | 1.005 (1.003,1.008) * | 1.006 (1.004,1.009) * |
| HW12×PM_2.5_ | 1.008 (1.006,1.010) * | 1.009 (1.006,1.012) * | 1.009 (1.005,1.012) * |
| HW13×PM_2.5_ | 1.005 (1.003,1.006) * | 1.006 (1.004,1.008) * | 1.005 (1.003,1.008) * |
| HW14×PM_2.5_ | 1.009 (1.006,1.011) * | 1.010 (1.006,1.014) * | 1.008 (1.004,1.013) * |
| HW15×PM_2.5_ | 1.013 (1.010,1.017) * | 1.014 (1.009,1.020) * | 1.013 (1.007,1.019) * |
| CS01×PM_2.5_ | 1.000 (1.000,1.000) * | 1.000 (1.000,1.001) * | 1.000 (0.999,1.001) |
| CS02×PM_2.5_ | 1.000 (1.000,1.000) * | 1.000 (1.000,1.001) * | 1.000 (0.999,1.001) |
| CS03×PM_2.5_ | 1.000 (1.000,1.001) * | 1.000 (1.000,1.001) * | 1.000 (0.999,1.001) |
| CS04×PM_2.5_ | 1.000 (0.999,1.001) | 1.000 (0.999,1.001) | 0.999 (0.997,1.001) |
| CS05×PM_2.5_ | 1.000 (0.999,1.001) | 1.000 (0.999,1.002) | 0.999 (0.997,1.001) |
| CS06×PM_2.5_ | 1.001 (1.000,1.003) * | 1.001 (0.999,1.004) | 1.001 (0.998,1.004) |

Adjusted for gender, age, region, education, occupation, personal annual income, smoking, hypertension, diabetes, IOP, and PM_2.5_. *P<0.05.

**Table S5.** According to the subgroup of glaucoma family history, the OR (95% CI) for the association of glaucoma with heatwaves and cold spells.

|  | Yes | No | P value |
| --- | --- | --- | --- |
| HW01 | 1.009 (0.988,1.030) | 1.015 (1.011,1.019) | 0.58 |
| HW02 | 1.015 (0.983,1.047) | 1.021 (1.015,1.028) | 0.72 |
| HW03 | 1.025 (0.984,1.066) | 1.028 (1.020,1.036) | 0.89 |
| HW04 | 1.010 (0.987,1.033) | 1.017 (1.012,1.021) | 0.56 |
| HW05 | 1.021 (0.987,1.056) | 1.024 (1.018,1.031) | 0.86 |
| HW06 | 1.028 (0.982,1.075) | 1.033 (1.024,1.042) | 0.84 |
| HW07 | 1.008 (0.982,1.034) | 1.019 (1.013,1.024) | 0.42 |
| HW08 | 1.018 (0.978,1.060) | 1.029 (1.021,1.037) | 0.61 |
| HW09 | 1.029 (0.976,1.086) | 1.039 (1.028,1.050) | 0.73 |
| HW10 | 1.013 (0.977,1.050) | 1.025 (1.018,1.032) | 0.53 |
| HW11 | 1.037 (0.978,1.100) | 1.040 (1.029,1.051) | 0.92 |
| HW12 | 1.056 (0.976,1.142) | 1.057 (1.042,1.072) | 0.98 |
| HW13 | 1.023 (0.967,1.083) | 1.042 (1.031,1.054) | 0.54 |
| HW14 | 1.039 (0.945,1.142) | 1.067 (1.047,1.086) | 0.60 |
| HW15 | 1.066 (0.940,1.209) | 1.091 (1.065,1.117) | 0.74 |
| CS01 | 1.116 (1.031,1.208) | 1.002 (0.995,1.009) | 0.01* |
| CS02 | 1.134 (1.044,1.232) | 1.010 (1.001,1.018) | 0.01* |
| CS03 | 1.168 (1.058,1.289) | 1.018 (1.007,1.028) | 0.02* |
| CS04 | 1.233 (1.014,1.475) | 0.995 (0.973,1.016) | 0.06 |
| CS05 | 1.131 (0.974,1.315) | 1.015 (0.996,1.034) | 0.21 |
| CS06 | 1.138 (1.130,1.693) | 1.041 (1.014,1.068) | 0.53 |

Adjusted for gender, age, region, education, occupation, personal annual income, smoking, hypertension, diabetes, IOP, and PM_2.5_. *P < 0.05.

**Table S6.** According to the subgroup of glaucoma family history, the OR (95% CI) for the association of PACG with heatwaves and cold spells.

|  | Yes | No | P value |
| --- | --- | --- | --- |
| HW01 | 1.001 (0.976,1.026) | 1.018 (1.011,1.025) | 0.20 |
| HW02 | 1.001 (0.964,1.040) | 1.026 (1.016,1.036) | 0.21 |
| HW03 | 1.009 (0.961,1.059) | 1.033 (1.021,1.045) | 0.36 |
| HW04 | 1.001 (0.974,1.029) | 1.020 (1.013,1.027) | 0.19 |
| HW05 | 1.008 (0.968,1.050) | 1.029 (1.019,1.039) | 0.33 |
| HW06 | 1.010 (0.956,1.067) | 1.040 (1.026,1.054) | 0.31 |
| HW07 | 0.955 (0.963,1.028) | 1.022 (1.014,1.031) | 0.07 |
| HW08 | 1.000 (0.951,1.051) | 1.036 (1.023,1.049) | 0.17 |
| HW09 | 1.003 (0.937,1.074) | 1.047 (1.031,1.063) | 0.23 |
| HW10 | 0.994 (0.949,1.041) | 1.029 (1.018,1.039) | 0.15 |
| HW11 | 1.006 (0.934,1.085) | 1.047 (1.031,1.064) | 0.31 |
| HW12 | 1.026 (0.930,1.133) | 1.068 (1.046,1.092) | 0.44 |
| HW13 | 0.992 (0.920,1.069) | 1.052 (1.035,1.069) | 0.13 |
| HW14 | 0.998 (0.880,1.132) | 1.080 (1.050,1.110) | 0.23 |
| HW15 | 1.028 (0.872,1.211) | 1.120 (1.061,1.145) | 0.33 |
| CS01 | 1.088 (1.003,1.180) | 1.002 (0.992,1.012) | 0.06 |
| CS02 | 1.122 (1.024,1.228) | 1.010 (0.998,1.021) | 0.04* |
| CS03 | 1.131 (1.022,1.251) | 1.014 (0.999,1.028) | 0.06 |
| CS04 | 1.149 (0.958,1.378) | 0.980 (0.951,1.010) | 0.14 |
| CS05 | 1.085 (0.934,1.260) | 0.997 (0.971,1.023) | 0.31 |
| CS06 | 1.302 (1.060,1.599) | 1.027 (0.991,1.064) | 0.08 |

Adjusted for gender, age, region, education, occupation, personal annual income, smoking, hypertension, diabetes, IOP, and PM_2.5_. *P < 0.05.

**Table S7.** According to the subgroup of glaucoma family history, the OR (95% CI) for the association of POAG with heatwaves and cold spells.

|  | Yes | No | P value |
| --- | --- | --- | --- |
| HW01 | 1.047 (0.983,1.115) | 1.018 (1.010,1.025) | 0.40 |
| HW02 | 1.083 (0.982,1.195) | 1.026 (1.015,1.037) | 0.32 |
| HW03 | 1.116 (0.987,1.263) | 1.033 (1.020,1.046) | 0.27 |
| HW04 | 1.059 (0.989,1.134) | 1.019 (1.012,1.027) | 0.30 |
| HW05 | 1.104 (0.991,1.231) | 1.029 (1.018,1.041) | 0.25 |
| HW06 | 1.145 (0.992,1.322) | 1.037 (1.022,1.054) | 0.24 |
| HW07 | 1.072 (0.992,1.159) | 1.020 (1.011,1.029) | 0.24 |
| HW08 | 1.127 (0.992,1.280) | 1.031 (1.017,1.045) | 0.22 |
| HW09 | 1.181 (1.002,1.391) | 1.043 (1.025,1.061) | 0.21 |
| HW10 | 1.115 (0.995,1.249) | 1.028 (1.016,1.040) | 0.21 |
| HW11 | 1.256 (1.001,1.575) | 1.047 (1.028,1.065) | 0.21 |
| HW12 | 1.275 (1.007,1.613) | 1.060 (1.035,1.086) | 0.23 |
| HW13 | 1.247 (1.013,1.534) | 1.042 (1.022,1.062) | 0.17 |
| HW14 | 1.306 (1.011,1.687) | 1.061 (1.028,1.095) | 0.23 |
| HW15 | 1.349 (1.000,1.821) | 1.087 (1.045,1.131) | 0.30 |
| CS01 | 1.189 (0.967,1.463) | 1.010 (0.996,1.025) | 0.19 |
| CS02 | 1.185 (0.967,1.452) | 1.020 (1.004,1.036) | 0.22 |
| CS03 | 1.408 (0.967,2.049) | 1.036 (1.014,1.058) | 0.26 |
| CS04 | 1.597 (0.853,2.993) | 1.000 (0.961,1.040) | 0.39 |
| CS05 | 1.519 (0.805,2.867) | 1.015 (0.980,1.052) | 0.44 |
| CS06 | 1.984 (0.880,4.474) | 1.067 (1.017,1.120) | 0.49 |

Adjusted for gender, age, region, education, occupation, personal annual income, smoking, hypertension, diabetes, IOP, and PM_2.5_.

**Table S8.** Sensitivity analysis for exclusion of 3927 cataract patients.

|  | Glaucoma | PACG | POAG |
| --- | --- | --- | --- |
| HW01 | 1.013 (1.008,1.018) | 1.017 (1.009,1.024) | 1.016 (1.008,1.024) |
| HW02 | 1.019 (1/012,1.026) | 1.025 (1.014,1.036) | 1.023 (1.011,1.035) |
| HW03 | 1.025 (1.016,1.034) | 1.032 (1.018,1.045) | 1.030 (1.016,1.045) |
| HW04 | 1.015 (1.010,1.020) | 1.019 (1.011,1.027) | 1.017 (1.009,1.026) |
| HW05 | 1.022 (1.014,1.030) | 1.028 (1.016,1.040) | 1.026 (1.014,1.039) |
| HW06 | 1.029 (1.019,1.040) | 1.039 (1.023,1.054) | 1.033 (1.016,1.050) |
| HW07 | 1.016 (1.010,1.022) | 1.021 (1.012,1.030) | 1.018 (1.008,1.028) |
| HW08 | 1.026 (1.016,1.035) | 1.034 (1.020,1.049) | 1.027 (1.012,1.043) |
| HW09 | 1.035 (1.023,1.047) | 1.044 (1.026,1.063) | 1.038 (1.017,1.058) |
| HW10 | 1.022 (1.013,1.030) | 1.026 (1.014,1.038) | 1.025 (1.012,1.039) |
| HW11 | 1.035 (1.023,1.048) | 1.044 (1.026,1.062) | 1.042 (1.021,1.063) |
| HW12 | 1.052 (1.035,1.069) | 1.067 (1.041,1.094) | 1.055 (1.027,1.084) |
| HW13 | 1.036 (1.023,1.049) | 1.049 (1.030,1.068) | 1.035 (1.013,1.058) |
| HW14 | 1.058 (1.036,1.081) | 1.079 (1.045,1.114) | 1.053 (1.017,1.091) |
| HW15 | 1.083 (1.053,1.114) | 1.106 (1.059,1.155) | 1.079 (1.033,1.128) |
| CS01 | 1.004 (0.996,1.012) | 1.003 (0.992,1.014) | 1.010 (0.993,1.027) |
| CS02 | 1.012 (1.003,1.022) | 1.012 (0.999,1.026) | 1.023 (1.003,1.043) |
| CS03 | 1.020 (1.008,1.032) | 1.014 (0.998,1.030) | 1.044 (1.016,1.072) |
| CS04 | 0.993 (0.969,1.019) | 0.971 (0.938,1.006) | 1.002 (0.955,1.051) |
| CS05 | 1.012 (0.990,1.033) | 0.985 (0.957,1.015) | 1.022 (0.981,1.065) |
| CS06 | 1.045 (1.014,1.076) | 1.026 (0.984,1.069) | 1.085 (1.023,1.150) |

Adjusted for gender, age, region, education, occupation, personal annual income, smoking, hypertension, diabetes, IOP, and PM_2.5_

**Table S9.** Sensitivity analysis for exclusion of 8498 hypertension patients and 2754 diabetes patients.

|  | Glaucoma | PACG | POAG |
| --- | --- | --- | --- |
| HW01 | 1.016 (1.011,1.022) | 1.020 (1.011,1.029) | 1.019 (1.010,1.028) |
| HW02 | 1.022 (1.014,1.030) | 1.027 (1.015,1.040) | 1.026 (1.013,1.039) |
| HW03 | 1.029 (1.019,1.039) | 1.035 (1.019,1.051) | 1.035 (1.019,1.050) |
| HW04 | 1.018 (1.012,1.024) | 1.022 (1.012,1.031) | 1.021 (1.011,1.030) |
| HW05 | 1.026 (1.018,1.035) | 1.031 (1.017,1.044) | 1.031 (1.017,1.045) |
| HW06 | 1.035 (1.023,1.046) | 1.042 (1.024,1.060) | 1.039 (1.021,1.058) |
| HW07 | 1.020 (1.013,1.027) | 1.024 (1.013,1.035) | 1.022 (1.011,1.033) |
| HW08 | 1.031 (1.021,1.042) | 1.038 (1.021,1.054) | 1.033 (1.017,1.050) |
| HW09 | 1.041 (1.028,1.055) | 1.046 (1.025,1.067) | 1.047 (1.025,1.069) |
| HW10 | 1.026 (1.017,1.035) | 1.028 (1.014,1.042) | 1.030 (1.016,1.045) |
| HW11 | 1.042 (1.028,1.055) | 1.046 (1.025,1.067) | 1.049 (1.027,1.072) |
| HW12 | 1.061 (1.042,1.080) | 1.070 (1.040,1.100) | 1.066 (1.036,1.097) |
| HW13 | 1.045 (1.031,1.060) | 1.053 (1.031,1.075) | 1.046 (1.022,1.070) |
| HW14 | 1.073 (1.048,1.098) | 1.079 (1.040,1.120) | 1.071 (1.032,1.112) |
| HW15 | 1.102 (1.069,1.135) | 1.101 (1.047,1.159) | 1.104 (1.054,1.157) |
| CS01 | 1.001 (0.993,1.010) | 0.998 (0.986,1.021) | 1.011 (0.994,1.027) |
| CS02 | 1.011 (1.001,1.021) | 1.007 (0.993,1.021) | 1.021 (1.003,1.040) |
| CS03 | 1.018 (1.005,1.030) | 1.009 (0.992,1.026) | 1.040 (1.015,1.065) |
| CS04 | 0.998 (0.973,1.024) | 0.980 (0.946,1.016) | 1.008 (0.964,1.054) |
| CS05 | 1.014 (0.992,1.036) | 0.996 (0.965,1.027) | 1.022 (0.982,1.063) |
| CS06 | 1.044 (1.012,1.077) | 1.020 (0.977,1.065) | 1.082 (1.023,1.143) |

Adjusted for gender, age, region, education, occupation, personal annual income, smoking, hypertension, diabetes, IOP, and PM_2.5_.

**Table S10.** Sensitivity analysis for adjusting relative humidity and air pressure.

|  | Glaucoma | PACG | POAG |
| --- | --- | --- | --- |
| HW01 | 1.012 (1.006,1.017) | 1.010 (1.002,1.018) | 1.018 (1.008,1.027) |
| HW02 | 1.016 (1.008,1.023) | 1.014 (1.002,1.025) | 1.026 (1.012,1.039) |
| HW03 | 1.021 (1.012,1.030) | 1.018 (1.004,1.032) | 1.032 (1.015,1.048) |
| HW04 | 1.013 (1.008,1.019) | 1.012 (1.003,1.020) | 1.020 (1.010,1.029) |
| HW05 | 1.019 (1.011,1.027) | 1.016 (1.004,1.029) | 1.029 (1.015,1.043) |
| HW06 | 1.026 (1.016,1.037) | 1.015 (1.010,1.042) | 1.036 (1.017,1.055) |
| HW07 | 1.015 (1.009,1.022) | 1.013 (1.004,1.023) | 1.021 (1.009,1.032) |
| HW08 | 1.023 (1.013,1.033) | 1.021 (1.007,1.036) | 1.029 (1.013,1.046) |
| HW09 | 1.032 (1.020,1.045) | 1.030 (1.011,1.049) | 1.042 (1.020,1.064) |
| HW10 | 1.020 (1.012,1.028) | 1.016 (1.004,1.029) | 1.028 (1.013,1.043) |
| HW11 | 1.031 (1.019,1.044) | 1.030 (1.011,1.049) | 1.043 (1.021,1.066) |
| HW12 | 1.047 (1.029,1.065) | 1.046 (1.020,1.074) | 1.057 (1.027,1.088) |
| HW13 | 1.036 (1.023,1.049) | 1.038 (1.019,1.058) | 1.040 (1.017,1.063) |
| HW14 | 1.060 (1.038,1.083) | 1.063 (1.029,1.098) | 1.060 (1.022,1.099) |
| HW15 | 1.084 (1.054,1.114) | 1.080 (1.033,1.128) | 1.088 (1.039,1.140) |
| CS01 | 1.016 (1.007,1.025) | 1.013 (1.001,1.026) | 1.033 (1.014,1.052) |
| CS02 | 1.025 (1.014,1.036) | 1.022 (1.007,1.038) | 1.038 (1.018,1.059) |
| CS03 | 1.031 (1.019,1.044) | 1.024 (1.007,1.042) | 1.047 (1.023,1.071) |
| CS04 | 1.028 (0.996,1.061) | 1.012 (0.963,1.064) | 1.014 (0.959,1.073) |
| CS05 | 1.014 (0.992,1.036) | 1.008 (0.975,1.042) | 0.983 (0.943,1.024) |
| CS06 | 1.061 (1.030,1.094) | 1.049 (1.002,1.097) | 1.073 (1.017,1.132) |

Adjusted for gender, age, region, education, occupation, personal annual income, smoking, hypertension, diabetes, IOP, PM_2.5_, relative humidity, and air pressure.

**Supplemental References:**

1. Foster PJ, Buhrmann R, Quigley HA, Johnson GJ. The definition and classification of glaucoma in prevalence surveys. Br J Ophthalmol. Feb 2002;86(2):238-42. doi:10.1136/bjo.86.2.238

2. Foster PJ, Devereux JG, Alsbirk PH, et al. Detection of gonioscopically occludable angles and primary angle closure glaucoma by estimation of limbal chamber depth in Asians: modified grading scheme. Br J Ophthalmol. Feb 2000;84(2):186-92. doi:10.1136/bjo.84.2.186

3. Scheie HG. Width and pigmentation of the angle of the anterior chamber; a system of grading by gonioscopy. AMA Arch Ophthalmol. Oct 1957;58(4):510-2. doi:10.1001/archopht.1957.00940010526005

4. Liang YB, Friedman DS, Wong TY, et al. Rationale, design, methodology, and baseline data of a population-based study in rural China: the Handan Eye Study. Ophthalmic Epidemiol. Mar-Apr 2009;16(2):115-27. doi:10.1080/09286580902738159

5. Liang Y, Friedman DS, Zhou Q, et al. Prevalence and characteristics of primary angle-closure diseases in a rural adult Chinese population: the Handan Eye Study. Invest Ophthalmol Vis Sci. Nov 7 2011;52(12):8672-9. doi:10.1167/iovs.11-7480

6. Papadopoulos M, Cable N, Rahi J, Khaw PT, Investigators BIGES. The British Infantile and Childhood Glaucoma (BIG) Eye Study. Invest Ophthalmol Vis Sci. Sep 2007;48(9):4100-6. doi:10.1167/iovs.06-1350

7. Yeung HH, Walton DS. Clinical classification of childhood glaucomas. Arch Ophthalmol. Jun 2010;128(6):680-4. doi:10.1001/archophthalmol.2010.96

8. Puttaswamy SJ, Nguyen HM, Braverman A, Hu XF, Liu Y. Statistical data fusion of multi-sensor AOD over the Continental United States. Geocarto Int. Jan 2 2014;29(1):48-64. doi:10.1080/10106049.2013.827750

9. Randles CA, Da Silva AM, Buchard V, et al. The MERRA-2 Aerosol Reanalysis, 1980 - onward, Part I: System Description and Data Assimilation Evaluation. J Clim. Sep 2017;30(17):6823-6850. doi:10.1175/JCLI-D-16-0609.1
